# Water Hammer Phenomenon in Coronary Arteries: Scientific Basis for Diagnostic and Predictive Modeling with Acoustic Action Mapping

**DOI:** 10.3390/diagnostics15050553

**Published:** 2025-02-25

**Authors:** Khiem D. Ngo, Thach Nguyen, Huan Dat Pham, Hadrian Tran, Dat Q. Ha, Truong S. Dinh, Imran Mihas, Mihas Kodenchery, C. Michael Gibson, Hien Q. Nguyen, Thang Nguyen, Vu T. Loc, Chinh D. Nguyen, Hoang Anh Tien, Ernest Talarico, Marco Zuin, Gianluca Rigatelli, Aravinda Nanjundappa, Quynh T. N. Nguyen, The-Hung Nguyen

**Affiliations:** 1 Department of Medicine, University of Texas Rio Grande Valley, Valley Baptist Medical Center, Harlingen, TX 78503, USA; luoikiemvang@gmail.com; 2 Interventional Cardiology, Methodist Hospital, Merrillville, IN 46410, USA; truongsdinh22@gmail.com (T.S.D.); drmihasmamu@yahoo.com (M.K.); nguyenquanghienmd@gmail.com (H.Q.N.); 3 Interventional Cardiology, St Mary Medical Center, Hobart, IN 46342, USA; 4 School of Medicine, Tan Tao University, Long An 82000, Vietnam; triloc27@gmail.com; 5 Department of Medicine, Conemaugh Memorial Medical Center, Johnstown, PA 15905, USA; huandat123@gmail.com; 6 Department of Internal Medicine, Palisades Medical Center, North Bergen, NJ 07601, USA; tranhadrian@gmail.com; 7 Wayne State University School of Medicine Internal Medicine Residency Program, Trinity Health Oakland, Pontiac, MI 48201, USA; datquangha@gmail.com; 8 Undergraduate Program, Indiana University, Bloomington, IN 46202, USA; mihasimran@gmail.com; 9 Baim Institute of Clinical Research, Harvard Medical School, Boston, MA 02115, USA; 10 Phenikaa University Hospital, Hanoi 100000, Vietnam; anh3tue@gmail.com; 11 Stroke International Service Hospital, Can Tho 900000, Vietnam; chinh0208@gmail.com; 12 Hue University of Medicine and Pharmacy, Hue University, Cardiovascular Centre, Hue 53000, Vietnam; bsanhtien@gmail.com; 13 Department of Biological Sciences, Purdue University Northwest, Hammond, IN 46323, USA; talaricojrernest@gmail.com; 14 Department of Translational Medicine, University of Ferrara, 44121 Ferrara, Italy; zuinml@yahoo.it; 15 Interventional Cardiology Unit, Division of Cardiology, Ospedali Riuniti Padova Sud, AULSS6, 35043 Padova, Italy; jackyheart@libero.it; 16 Peripheral Interventions, Cardiovascular Department, Cleveland Clinics Main Campus, Cleveland, OH 44195, USA; nanjuna@ccf.org; 17 AISIA Research Laboratories, University of Science, Vietnam National University, Ho Chi Minh 700000, Vietnam; thuynguyetquynh@gmail.com; 18 Vietnam Association for Fluid Mechanics and Water Resources Engineering Department, University of Science and Technology, The University of Danang, Danang 550000, Vietnam; ngthung@dut.udn.vn

**Keywords:** coronary lesion, fluid mechanics, compression zone, rarefaction zone, pressure wave reflection, acoustics, water hammer, antinode, nodes, coronary acoustic action map

## Abstract

**Background:** In the study of coronary artery disease, the mechanisms underlying atherosclerosis initiation and progression or regression remain incompletely understood. Our research conceptualized the cardiovascular system as an integrated network of pumps and pipes, advocating for a paradigm shift from static imaging of coronary stenosis to dynamic assessments of coronary flow. Further review of fluid mechanics highlighted the water hammer phenomenon as a compelling analog for processes in coronary arteries. **Methods:** In this review, the analytical methodology employed a comprehensive, multifaceted approach that incorporated a review of fluid mechanics principles, in vitro acoustic experimentation, frame-by-frame visual angiographic assessments of in vivo coronary flow, and an artificial intelligence (AI) protocol designed to analyze the water hammer phenomenon within an acoustic framework. In the analysis of coronary flow, the angiograms were selected from patients with unstable angina if they had previously undergone one or more coronary angiograms, allowing for a longitudinal comparison of dynamic flow and phenomena. **Results:** The acoustic investigations pinpointed pockets of contrast concentrations, which might correspond to compression and rarefaction zones. Compression antinodes were correlated to severe stenosis, due to rapid shifts from low-pressure diastolic flow to high-pressure systolic surges, resulting in intimal injury. Rarefaction antinodes were correlated with milder lesions, due to de-escalating transitions from high systolic pressure to lower diastolic pressure. The areas of nodes remained without lesions. Based on the locations of antinodes and nodes, a coronary acoustic action map was constructed, enabling the identification of existing lesions, forecasting the progression of current lesions, and predicting the development of future lesions. **Conclusions:** The results suggested that intimal injury was likely induced by acoustic retrograde pressure waves from the water hammer phenomenon and developed new lesions at specifically exact locations.

## 1. Introduction

The mechanisms underlying the initiation of atherosclerosis and its progression or regression remain poorly understood. Existing coronary imaging modalities and physiological assessments (intravascular ultrasound, optical coherence tomography, fractional flow reserve, etc.) are insufficient for providing definitive etiological insights or comprehensive prognostic evaluations [1]. To address these gaps, our research conceptualized the cardiovascular system as a unified network of pumps and pipes, advocating for a paradigm shift from static imaging of coronary luminal stenosis to dynamic assessments of coronary flow [2]. Leveraging principles of fluid mechanics, we investigated abnormal flow patterns that disrupt the integrity of the endothelium in vascular and pump-like components. Specifically, we focused on characterizing flow disruptions—including recirculating, antegrade, retrograde, and disorganized flows, as well as their collisions—which potentially trigger the atherosclerotic cascade [3].

As further review progressed, it was evident that the damage to the intimal layer could not be solely attributed to a simplistic fluid mechanics interaction between antegrade and retrograde flows. A more detailed review of specialized fluid mechanics literature and practice was essential to identify the water hammer phenomenon as potential mechanism capable of causing significant structural disruption. Could a similar abnormal flow occur within coronary arteries when analyzed through a more advanced fluid mechanics framework such as acoustics? What coronary imaging evidence could support its existence, and what implications might it have for coronary arterial integrity and functionality?

To promote innovation and dynamism, this combined investigative format diverges from traditional literature reviews by employing a novel approach. By establishing a comprehensive theoretical and experimental framework, it aimed to develop advanced diagnostic methodologies and predictive models. Computational and conceptual simulations were utilized to examine coronary artery dynamics under specific conditions, including the water hammer phenomenon. Furthermore, the integration of sound-based techniques to map and visualize interactions between acoustic signals and arterial structures provided critical insights into the initiation, progression, and regression of coronary lesions. By combining angiographic imaging analysis, computational modeling, and acoustic mapping, this analysis–investigation review aimed to build a strong scientific foundation for the search for the mechanism driving the atherosclerotic process and to elucidate unresolved clinical challenges, including unpredictable plaque rupture and the silent progression of lesions.

This review investigation format was systematically organized into a four-phase framework. The first phase concentrated on defining the fundamental principles governing fluid mechanics in pipe systems, establishing a robust foundation for identifying similar critical parameters relevant to coronary flow dynamics. The second phase integrated acoustic principles to address the limitations of conventional fluid mechanics and enhance the precision of key coronary parameter identifications and quantifications through in vitro analysis of air particle motion within a tubular system. The third phase applied these insights to investigations of in vivo coronary flow, focusing on acoustic mechanisms implicated in the formation of new and growth of existing lesions. The final phase synthesized these insights to establish novel standards for coronary physiology and pathophysiology while proposing practical clinical and interventional solutions rooted in fluid mechanics and acoustics. These advancements are designed for application both at the bedside and within cardiac catheterization or interventional laboratories.

While clinician–scientists and fluid mechanics engineers conducted in vitro experiments and in vivo coronary angiographic analyses, the data science team utilized artificial intelligence (AI) and machine learning (ML) algorithms to independently analyze contrast dynamics in coronary arteries. Their objective was to establish an AI-driven protocol capable of real-time image analysis, alleviating the need for labor-intensive, frame-by-frame evaluations traditionally performed by overburdened interventional cardiologists.

**Figure 1:**
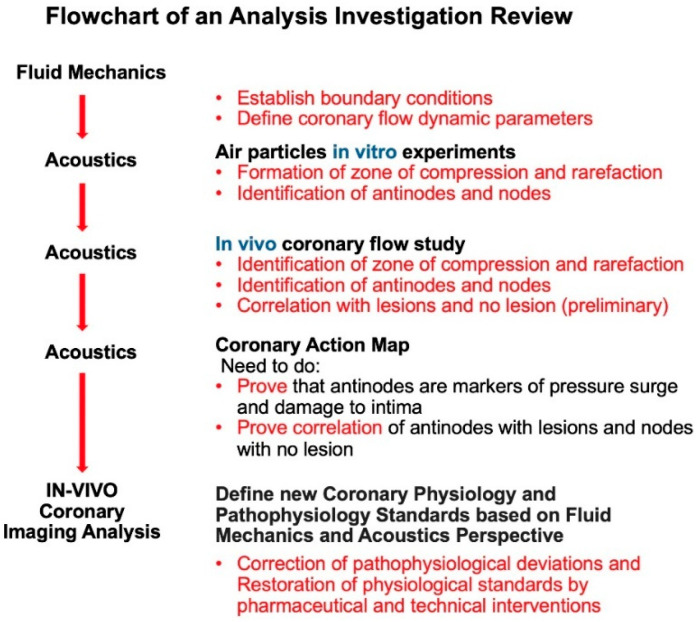
Flowchart of the analysis-combined-investigation review.

To foster in-depth and thought-provoking discussions among clinician–scientists, cardiologists, and fluid mechanics engineers, each major section—spanning methodology, fluid mechanics analyses, and acoustic investigations—was concluded with a “Critical Thinking” summary. These summaries distilled key insights from fluid mechanics and acoustics, identified unresolved questions related to coronary dynamics and flow phenomena, and underscored the need for further exploratory research and innovative clinical applications. Particular attention was given to integrating this dynamic flow-based approach with traditional imaging modalities and physiologic assessments. The flowchart of this interactive analysis-combined-investigation review is presented in Figure 1.

## 2. Methods

To establish a scientific basis for potential manifestations of major fluid mechanics phenomena in coronary arteries, we began by evaluating the strengths and limitations of existing imaging techniques [4]. Leveraging insights from our novel dynamic flow studies, based on the coronary angiographic images of a small group of patients, we analyzed distinct flow patterns, including antegrade and retrograde flows, as well as their interactions from a general fluid mechanics perspective [3]. However, characterizing these interactions solely as “collisions” was inadequate to fully elucidate the mechanisms underlying the development and progression of coronary lesions. To overcome this limitation, a comprehensive review of advanced fluid mechanics literature highlighted the water hammer phenomenon as a promising concept with potential parallels to processes occurring in coronary arteries [5]. Accordingly, the analytical methodology employed a comprehensive, multifaceted approach that incorporated a review of fluid mechanics principles, in vitro acoustic experimentation, frame-by-frame visual angiographic assessments of in vivo coronary flow, and an artificial intelligence (AI) protocol designed to analyze the water hammer phenomenon within an acoustic framework [6,7,8].

**Patient Population.** From January 2020 to December 2024, patients coming to the cardiac catheterization laboratories (CCL) for outpatient coronary angiography with diagnosis of unstable angina were screened for inclusion. Patients were included in the study if they had previously undergone one or more coronary angiograms, allowing for a longitudinal comparison of dynamic flow and phenomena between two or more angiograms. Patients with history of prior percutaneous coronary intervention (PCI) or coronary artery bypass graft surgery (CABG) were excluded. Because the goal of this analysis investigation review was to set a scientific basis for using acoustics in the study of coronary arteries, only the preliminary qualitative characteristics of the coronary flows of a small number of patients are presented.

**Figure 2:**
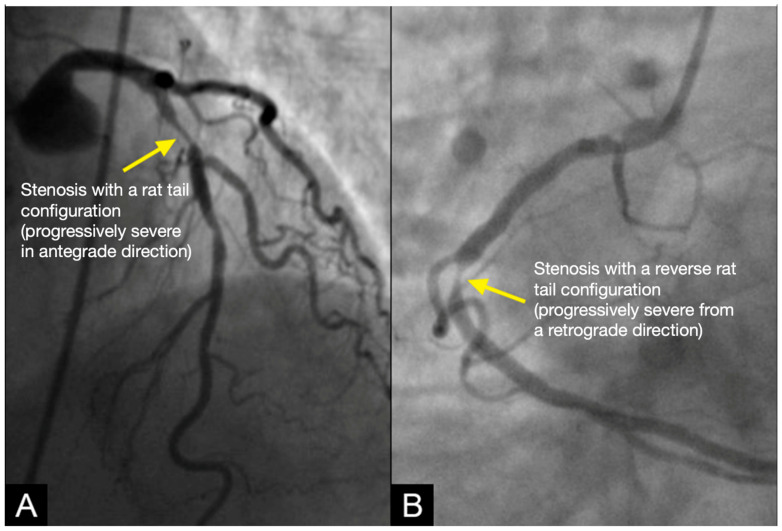
The left anterior descending (LAD) artery exhibits severe narrowing in its proximal segment (indicated by an arrow), while the right coronary artery (RCA) demonstrates a subtotal lesion in its mid-segment. Current angiographic techniques capture these lesions but fail to provide critical insights into their formation mechanisms or potential progression over time. (**A**) The lesion in the proximal LAD has a characteristic “rat tail” appearance—featuring progressively severe narrowing in the distal direction. (**B**) The mid-RCA lesion has a “reversed rat tail” pattern, characterized by more severe narrowing proximally and a gradual reduction in severity distally. WHY?

**Conventional Coronary Imaging**. Currently, angiography is considered the gold standard for the diagnosis of coronary artery disease, providing detailed visualization of coronary luminal stenosis (Figure 2) [3]. However, angiography is limited to static imaging, offering no insights into the dynamic processes driving development of lesions, the mechanism of their anatomical distribution, or the factors governing their progression or regression.

**Physiological Measurements by Intravascular Ultrasound and Optical Coherence Tomography.** Abnormal wall shear stress (WSS) is a well-established contributor to the development of atherosclerosis; however, its accurate quantification presents significant challenges. Current approaches primarily rely on invasive techniques such as intravascular ultrasound and optical coherence tomography, which are not only invasive but also costly, technically demanding, and challenging to standardize, thereby limiting their widespread use. Additionally, direct measurement of WSS under physiological conditions is impractical, and a standardized protocol for deriving WSS from cardiac computed tomography angiography data has yet to be established [9,10]. WSS has been studied for over 75 years; however, this investigative methodology has yet to yield conclusive or disruptive results [11]. Consequently, WSS was not prioritized as the primary method of investigation in this analysis investigation review.

**Figure 3:**
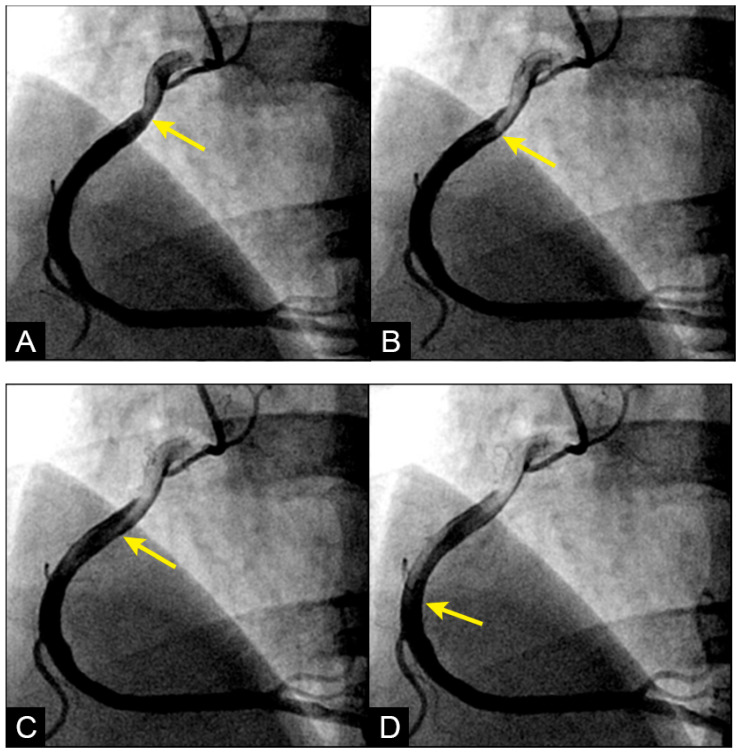
(**A**–**D**) **Laminar flow**. These four coronary images are of consecutive sequence. (**A**) This is the angiogram of the right coronary artery (RCA), which is filled with contrast in black. (**B**) The blood (in white) is seen well organized with sharp border and a pointed tip, typical for laminar flow, moving in (yellow arrow). (**C**,**D**) The blood is seen following the apex of the curves (yellow arrow). This is the laminar flow following the curves in a helical fashion.

**Dynamic Coronary Angiography.** In a shift in strategy, our research adopted a novel approach by conceptualizing the cardiovascular system as an interconnected network of pipes and pumps, paving the way for the development of an innovative angiographic technique capable of capturing real-time coronary flow dynamics [2]. Drawing experiences from fluid mechanic engineering, where flow disturbances compromised the integrity of pipe surfaces, we hypothesized that analogous forces may damage the arterial intima. Accordingly, our analysis emphasized the characterization of blood flow—whether laminar, recirculating, disorganized—and its correlation with the initiation, progression, or regression of coronary lesion as seen in Figure 3 [3].

This approach defines baseline flow as antegrade and laminar, with deviations considered abnormal. Retrograde flow colliding with antegrade flow is a primary mechanism of vascular injury. A thick lateral contrast layer may indicate prolonged, subtle intimal damage, potentially leading to gradual lesion formation. Laminar flow supports long-term coronary patency.

**Fluid Mechanics Analysis**. The attempt to substantiate the occurrence of the water hammer event in coronary arteries required addressing dual tactical challenges unique to this context. In addition to understanding the fluid mechanics mechanisms and implications of water hammer effects in pipes, it was crucial to identify similar flow characteristics in coronary arteries, including their underlying mechanisms, lesion locations, boundary conditions, and dynamics of pressure wave reflections within the constraints of the relatively short coronary arteries compared to long domestic pipes or industrial pipelines [6]. Table 1 outlines the key dual logistic challenges of fluid mechanics in tubes and angiographic flow dynamics in arteries that must be addressed to advance this analysis investigation.

**Figure 4:**
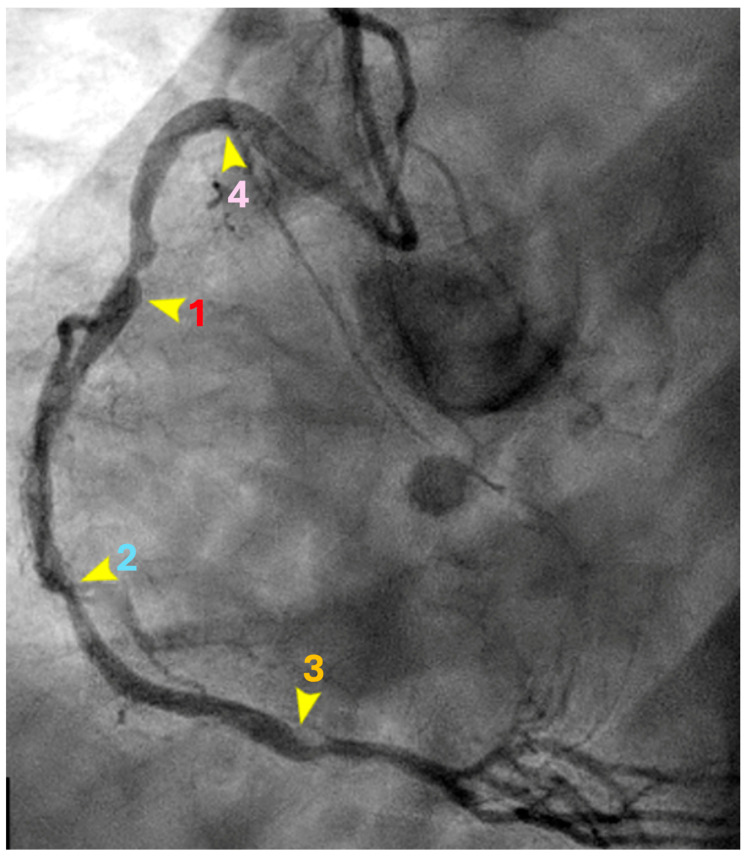
This is the angiogram of the right coronary artery. The lesions are at locations **1**, **2**, and **3**. Why is the lesion at 1 more severe than the ones at **2** and **3**? Why is the lesion at 2 less severe than **1** and **3**? Why is there no lesion in **4**? Could fluid mechanics and acoustics explain the mechanism of formation and growth of the lesions at these specific locations?

**Acoustic Investigations**. Once the boundary conditions and flow dynamics in coronary arteries were established through simulations replicating analogous processes in pipes, the investigation shifted to analyzing pressure wave reflections [7]. To explore this phenomenon, acoustic principles were incorporated to examine flows propagating near the speed of sound in both pipes and coronary arteries. Building on the principles of sound wave propagation—where particle displacement creates alternating zones of compression and rarefaction—we hypothesized that these acoustic phenomena could be applied to coronary angiograms to delineate distinct zones of high-intensity contrast (compression zones, also called as antinodes), moderate-density contrast (rarefaction zones, also categorized as antinodes), and minimal contrast concentration (nodes). These zones of contrast concentration were interpreted as surrogates for varying pressure surge intensities capable of damaging the arterial intima. The antinodes were subsequently analyzed for their potential association with lesion development and progression, while the nodes were examined as regions exhibiting minimal or absent lesions [8]. The goals of the investigation of coronary flow dynamics based on acoustics practice are listed in Figure 4 and Table 2.

**New Protocol of Coronary Images Analysis.** When analyzing coronary angiographic images, it is crucial to select a projection that provides an unobstructed longitudinal view of the entire artery, free from superimposition by other arteries, branches, the liver, or shadows from spinal vertebrae. After the artery is fully opacified in black by the contrast agent, and as the contrast injection ceases while recording continues, the incoming blood appears in white as it displaces the contrast. This sequence enables the observation of flow dynamics as the blood progressively replaces the contrast agent (Figure 3A–D) [2]. By the end of the sequence, when most of the contrast has been replaced by blood, isolated pockets of contrast stagnation remain. These stagnant pockets might represent the zones and mechanism of high concentration of contrast (compression) or moderate concentration of contrast (rarefaction) as a result of passage by the water hammer-generated pressure wave (Figure 4).

**Figure 5:**
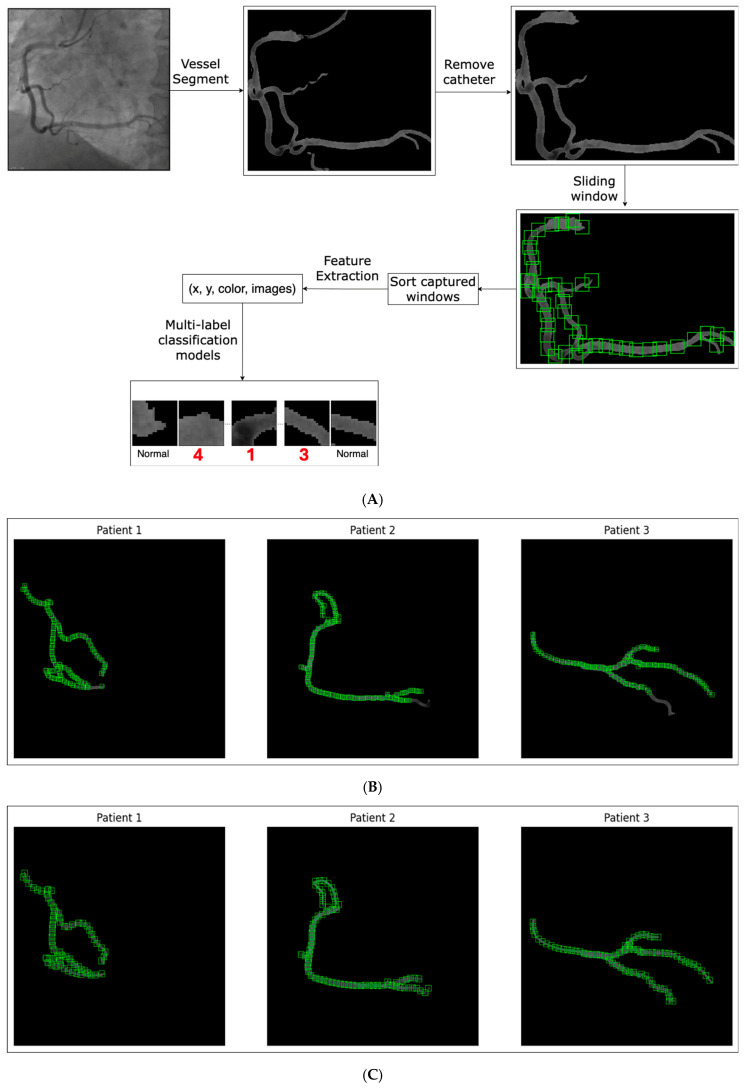

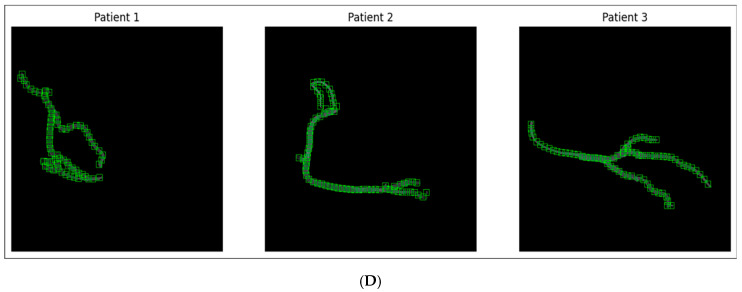
(**A**) Artificial intelligence algorithm to segment the arteries and the catheter. (**B**) Window size = 10 pixels. (**C**) Window size = 15 pixels. (**D**) Window size = 20 pixels.

**Artificial Intelligence PROTOCOL**. Artificial intelligence algorithms to independently analyze the contrast movements in coronary arteries to detect nodes: 1, 2, 3, and 4. For this task, the R2U Attention architecture to segment vessels was used [12] (Figure 5A–D).

Two models were trained: one for segmenting heart vessels, and the other for segmenting catheters. To achieve accurate vessel segmentation, the results of these two models were subtracted. This step was crucial as it decided the overall accuracy of the process. To identify the antinodes (**1**, **2**, **3**, and **4**), a sliding window technique that slid across the vessel segmentation was used. The window size, currently set at 20 × 20 pixels, was tuned to fit most vessel segmentations. For each window, its coordinates on the image were captured, the sum of pixel values was calculated to represent the area’s intensity, and a small image was extracted from the window.

Similar to our previous work in stenosis detection [3], we used the Vision Transformer architecture [13] combined with the Two-Way Loss function [14], a loss function designed for multi-label classification, to classify nodes **1**, **2**, **3**, and **4**. The input was features captured from the sliding window step, while the output was the coordination of nodes. For each node type, a separate model was trained. Thus, for three node types, we had three distinct models to detect all nodes.

**CRITICAL THINKING on Methodology.** At the outset, the literature review (including in silico studies) concluded that the development and progression of arterial lesions could not be adequately explained by simplistic interpretations of flow collision rooted in basic fluid mechanics. Consequently, our research strategy prioritized examining the intricate acoustic effects arising from water hammer pressure wave reflections, with a particular focus on their potential analogs in coronary arteries. To enhance the likelihood of meaningful insights, this investigation of atherosclerosis mechanisms adopted an interdisciplinary framework, integrating concepts from fluid mechanics, in vitro acoustics experiments, in vivo coronary flow dynamics, and AI algorithm analysis within an acoustic paradigm.

## 3. Water Hammer Phenomenon

**Figure 6:**
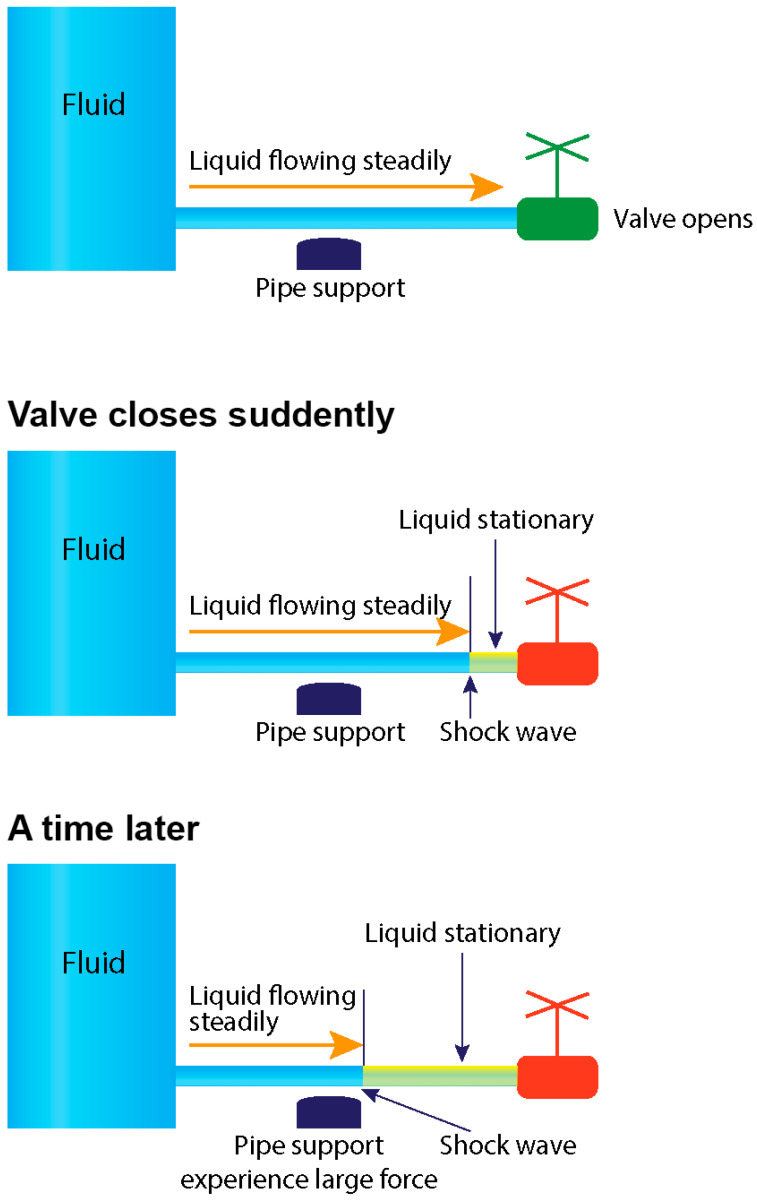
A water hammer event occurs when there is an abrupt change in velocity or flow direction in the pipe systems such as power failure, pump start-up, and shut-down operations as a pressure wave propagates backward in the pipe [2].

The water hammer event, a prevalent cause of damage in hydraulic systems, arises when the forward flow is abruptly interrupted by the sudden closure of a valve or the rapid stop of a pump [5]. This abrupt interruption generates a reflected pressure wave that propagates backward, colliding with the forward flow and producing a shockwave capable of damaging the inner surfaces of pipes and components of pump (Figure 6).

**Strategic Plan.** The investigation of water hammer phenomena in coronary arteries was organized into three progressively intricate and focused sections. The first section conducted a comprehensive review of the literature, drawing on principles of fluid mechanics and practical engineering case studies to elucidate the mechanisms of damage in pipe induced by the water hammer event. This foundational analysis defined critical boundary conditions and characterized flow dynamics under varied scenarios, including long pipes, short arterial segments, rigid structures, and arteries with flexible or calcified walls [15]. The second section examined in vitro particle motion within tubular models, employing acoustics methodologies to assess their dynamic interactions [16]. Lastly, the third section investigated in-vivo flow dynamics and transport of contrast agents within coronary arteries, juxtaposing these findings with particle motion observed in the tubular models. This comparative approach facilitated a deeper understanding of the interplay between lesion development and flow patterns which were governed by acoustic principles.

## 4. Fluid Mechanics Analysis

**MECHANISMS and LOCALIZATION. Fluid Mechanics Perspective.** In pipes, damage from a water hammer event typically originates first at the points where the pressure wave reflects, often near valves, fittings, or abrupt changes in pipe diameter [15]. These directional shifts create stress concentrations on joints and connections, potentially leading to leaks, wall cracks, or even rupture, depending on the magnitude of the pressure surge [15]. The second critical location is immediately upstream of the valve, where the highest pressure builds up. The third vulnerable area is at the points where the pipe is anchored or fixed to surrounding structures. Here, the retrograde wave interacts with rigid supports, causing localized pressure spikes and fatigue stress over time [15]. Finally, regions with compromised structural integrity—such as areas affected by corrosion, wall thinning, or prior repairs—are particularly susceptible to failure. These weakened sections may lack the resilience to withstand the abrupt pressure surge imposed by the retrograde wave, leading to potential structural collapse [15].

**Figure 7:**
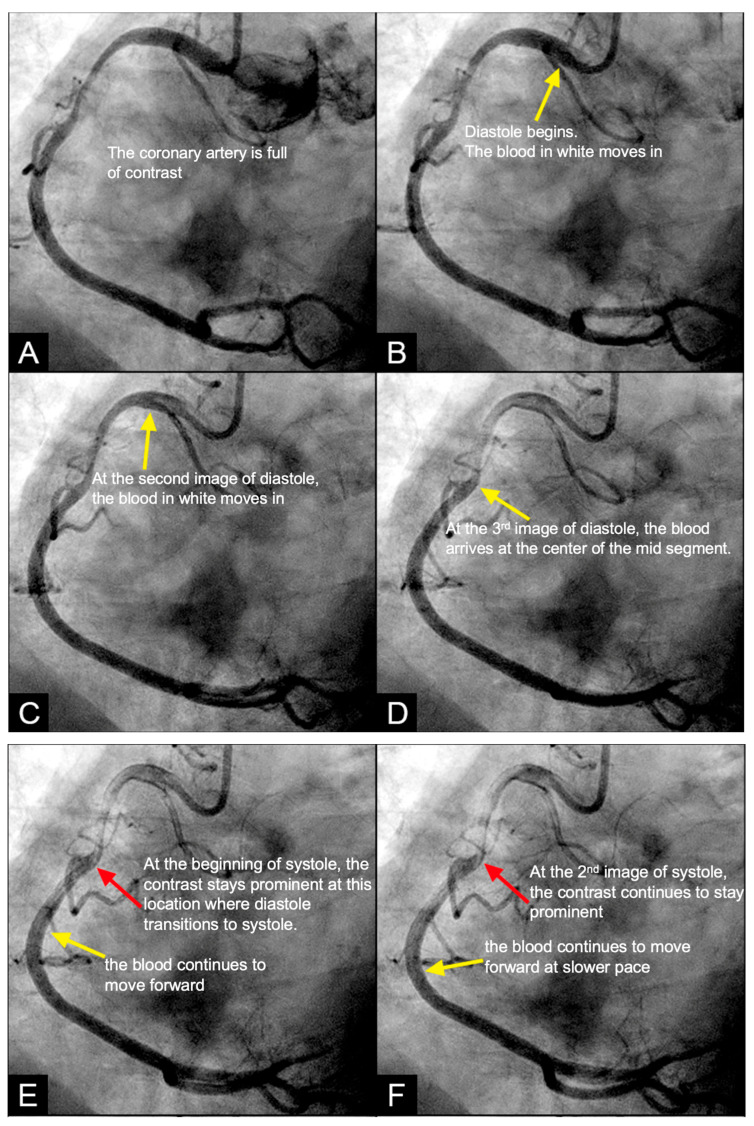

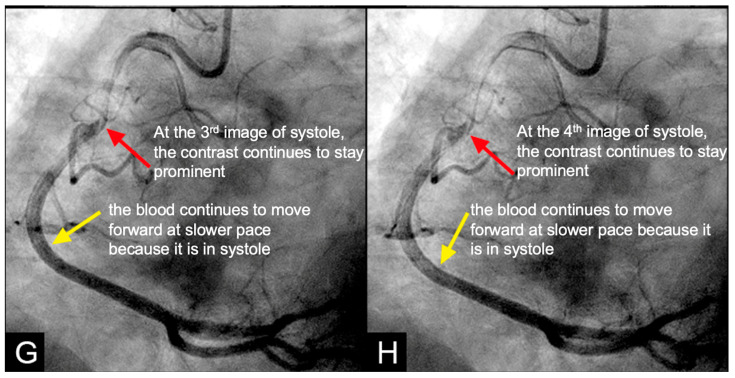
(**A**–**D**) **Collision in the right coronary artery**. This is a series of eight consecutive images of an angiogram of the right coronary artery (RCA) separated by a 0.067 s gap. (**A**) The artery is filled with contrasts. There is a moderate lesion at the mid-segment. (**B**) The blood (white) is seen entering the ostium of the RCA (arrow). This is the beginning of diastole. (**C**) The blood (white) is seen at the outer border of the first curve of the RCA (yellow arrow). (**D**) The blood (white) moves to the mid-segment of the RCA (yellow arrow). (**E**–**H**)**ollision during the transition from diastole to systole.** (**E**,**F**) The blood is seen reaching the mid-segment of the RCA (yellow arrow) at the end of diastole and beginning of systole. Here, the blood (white) is mixed with the contrast (black), seen as a random, disorganized flow (mixed of black and white) This is the visual image of disorganized, turbulent flow (red arrow). (**G**) The contrast (black) concentrates at the mid-segment, at the collision line (red arrow). The antegrade flow still moves forward slowly (yellow arrow). (**H**) The blood is seen reaching the beginning of the distal segment (yellow arrow). The turbulent flow (mixing black contrast and white blood) is still seen prominently at the collision site (red arrow).

**Cardiovascular Perspective**. During a water hammer event within a coronary artery, the retrograde pressure wave would reflect and collide with the antegrade flow, first at the leading edge of the advancing flow, which is located at the transition from diastole to systole (red arrows in Figure 7E–H). The damage mechanism involves the superposition of forward and backward pressure waves, producing a pronounced pressure spike that may induce microtears in the endothelial layer [17]. These microtears may facilitate the migration of low-density lipoprotein (LDL) cholesterol molecules into the subintimal space, if the serum LDL level is high, starting the atherosclerotic cascade [18]. Additionally, the rapid flow reversal may disrupt normal shear stress distribution, further compromising the integrity of endothelial cells and weakening their role as structural barrier [18]. This pressure surge may be a key factor in intimal damage, triggering the cascade of events leading to atherosclerosis. The critical challenge lies in identifying and evidencing these damage mechanisms through currently available coronary angiographic images.

**BOUNDARY Conditions.** In fluid mechanics, an “open end” in a pipe refers to an outlet exposed to atmospheric pressure, whereas a “closed end” denotes a termination not directly exposed to the atmosphere [19].

**Fluid Mechanics Perspective.** In the context of retrograde pressure wave reflections during a water hammer event, one end of the tube typically acts as the source of the pressure wave (e.g., a rapidly closing valve), while the opposite end serves as the reflecting surface. This reflecting surface may represent a closed pipe end, an abrupt change in pipe diameter, or even an open end. The pressure wave propagates from the source toward the reflecting surface and subsequently rebounds, generating the retrograde wave [5].

At the open end of a tube, the reflection of a pressure wave manifests as a rarefaction wave due to the pressure boundary condition imposed by the atmosphere. When a wave reaches the open end, the pressure at this boundary must align with atmospheric pressure because it is directly exposed to the surrounding air. If a compression wave, characterized by high pressure, travels toward the open end, the system reflects a wave of opposite nature—a rarefaction wave—to restore equilibrium. This process facilitates the system’s adjustment to the open boundary by dissipating the energy outward into the atmosphere, thereby minimizing the disturbance introduced by the incoming compression wave [19].

**Figure 8:**
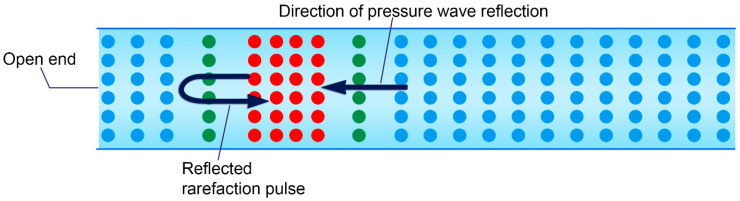
As the coronary artery is modeled as a tubular structure, the ostium of the coronary artery is an open end. As result, the flow will reverse as a rarefaction pulse, which has less concentrated particles.

**Cardiovascular Perspectives**. According to the above definition, in a water hammer event, the coronary artery was modeled as a tube with distinct proximal and distal ends. The proximal end is located within the distal coronary vasculature, where antegrade blood flow is interrupted by left ventricular contraction. This location represents a closed-end boundary because there is no direct connection with left ventricle. Conversely, the distal end, situated near the coronary ostium, functions as an open end. At this location, the flow encounters blood in the aortic root, disperses its particles to balance the two zones, returns as a rarefaction pressure wave toward the distal coronary vasculature (Figure 8) [20].

**SHORT TUBES and ARTERIES**. **Fluid Mechanic and Cardiovascular Perspective**. The coronary artery, with a relatively short length of 4–10 cm, requires a fluid mechanics analysis specific to the dynamics of short tubes. A key characteristic of such systems is the phenomenon of **rapid wave reflection**, wherein the limited length of the artery allows pressure waves to reach the distal end and reflect almost instantaneously. This rapid reflection minimizes the buildup of high-magnitude pressure surges, as the limited mass of fluid in motion within the short artery does not generate substantial pressure waves [21]. The second characteristic, **wave superposition**, arises from the proximity of the tube ends. Reflected waves quickly overlap with incoming waves, forming standing wave patterns and inducing transient pressure fluctuations, although these fluctuations are less intense than those observed in longer tubes [22].

**Figure 9:**
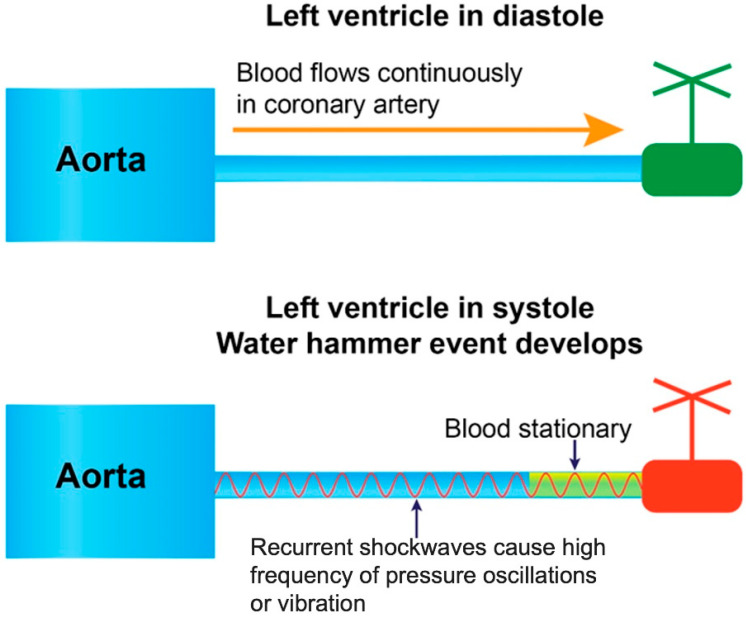
**Pressure wave reflections in short artery.** A water hammer event can occur in the coronary arteries when there is an abrupt change in velocity or flow direction due to sudden contraction of the left ventricle, creating a pressure shockwave reflecting at the speed of sound. The short length of a tube or an artery can result in a higher frequency of pressure oscillations, which is also called vibration.

In the fluid mechanic analysis in coronary artery, the third characteristic is **reduced peak pressure amplification**, as the constrained length restricts momentum transfer, leading to lower peak pressures compared to longer tubes where fluid velocity has more distance to build on before encountering a sudden stop [23]. This detail may explain why only long coronary artery develops lesion at its proximal segment, while short artery usually has no lesion. Finally, the short length of a tube or an artery can result in a **higher frequency of pressure oscillations**, as the brief travel time between tube ends facilitates rapid, multiple reflections, creating high-frequency oscillatory patterns (vibration) rather than singular, high-amplitude surges [24]. These features suggest that in the coronary arteries, pressure wave reflections during water hammer events are characterized by rapid, low-intensity oscillations with limited pressure buildup (Figure 9).

**FLEXIBLE versus RIGID ARTERIES**. **Cardiovascular Perspective**. In compliant arteries, pressure wave reflection enables the artery to expand and contract, facilitating the accommodation and transmission of the pressure wave with greater flexibility. This ability to undergo slight deformation helps reduce wave pressure, thereby mitigating localized pressure spikes and promoting a more gradual pressure profile along the vessel [25]. This may also explain the absence of coronary lesions in younger individuals with flexible arterial walls.

**Figure 10:**
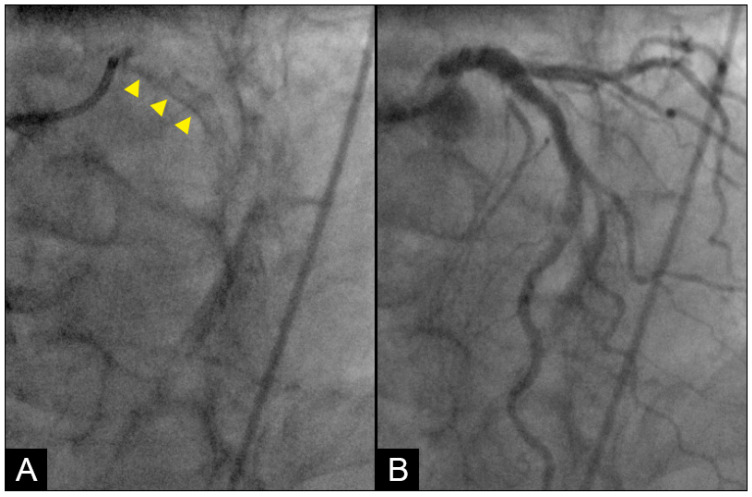
(**A**,**B**) **Calcification without flow limiting lesion.** (**A**) The proximal segment of the left anterior descending (LAD) artery was well calcified (arrows), while the mid and distal segment were spared. (**B**) In the same view, the angiogram of the proximal segment of the LAD shows no severe lesion. Prolonged mild to moderate turbulence without high cholesterol level causes only injuries to the intima, leading to heavy calcification without forming atherosclerotic plaques.

Conversely, in arteries affected by calcification, the vessel’s capacity to expand and contract becomes markedly compromised. The inability of the stiffened arterial wall to absorb the energy of the waves results in heightening the risk of endothelial microtears and further structural damage to the arterial wall (Figure 10) [26]. This phenomenon is particularly pronounced at the interface between calcified and non-calcified segments, which accounts for the frequent occurrence of new in-stent restenosis at the junction between the stent’s distal end and the arterial wall [27].

**Figure 11:**
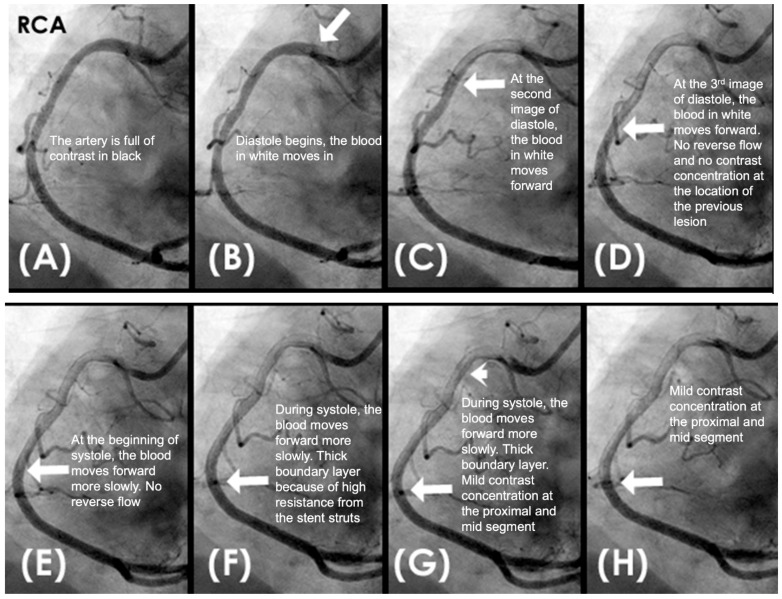
(**A**–**H**) This is a series of six consecutive images of an angiogram of the right coronary artery (RCA) right after stenting of the mid-segment. The baseline images before stenting are in Figure 7. (**A**) The artery is filled with contrasts. (**B**) The blood (white) is seen entering the ostium of the RCA (arrow). This is the beginning of diastole. (**C**–**H**) The blood (white) moves forward without encountering the retrograde pressure wave. Most likely the arterial wall is scaffolded by the stent which interrupts the propagation of retrograde pressure wave on the arterial wall.

Additionally, this process elucidates the mechanism driving plaque cover rupture, which often arises at the junction between the plaque and adjacent normal tissue due to the impact of pressure waves on the mechanically unequal interface. As arterial stiffening progresses with calcification, it impedes the retrograde propagation of pressure waves [28]. This underscores the beneficial role of stenting in mitigating retrograde pressure wave effects on the new stent-scaffolded arterial wall. (Figure 11A–H).

**CRITICAL THINKING on Coronary Flow Analysis from a Fluid Mechanics Perspective**. The fluid mechanic analysis of the water hammer event in coronary arteries examines potential analogies between the pressure spikes damaging pipe linings and the similar effects on arterial endothelial layers. This analysis considers the unique boundary conditions of coronary arteries, defined by a closed end at the contracting left ventricle and an open end at the coronary ostium, where flow interacts with the aortic root. Additionally, the discussion encompasses the role of pipe length and its arterial counterpart, emphasizing the exceptionally short length of coronary arteries, as well as the influence of material stiffness—whether intrinsic to pipes or due to calcification in arterial walls. These fluid mechanics parameters provide a framework for advancing the investigations of pressure wave reflection from an acoustic perspective with enhanced precision and insight.

## 5. Acoustic Investigations

**Strategic Plan.** The findings of our review into flow dynamics in pipes, combined with fluid mechanics analysis in coronary arteries, indicated that the retrograde pressure wave generated by a water hammer phenomenon, moving near to the speed of sound, might exert a pronounced impact on atherosclerosis initiation and progression [7]. This observation raised an intriguing question: how can acoustic principles and methodologies enhance our understanding of the interplay between pressure wave dynamics and development of lesions in coronary arteries?

To explore these questions, it was crucial to first investigate the in vitro dynamics of air particles within a confined tube, starting with their baseline distribution and subsequent motion in response to the propagation of a retrograde pressure wave. How did these particles interact with the wave? Did they cluster in specific regions while avoiding others? What mechanisms drove these distribution patterns?

Expanding these observations to coronary arteries introduced additional questions regarding the dynamics of contrast medium injected into these vessels. The movement of contrast in coronary artery might exhibit parallels to the motion of air particles, potentially correlating pockets of contrast concentration with the localization of coronary lesions. Once the pockets of contrast concentration were highlighted with correlated lesions, an acoustic action map could be drawn, serving as a roadmap for identifying both current and prospective lesions. The goals of the investigation about the relationships among contrast movement, coronary flow dynamics, and stenotic lesions from an acoustic perspective are summarized in Table 3.

**Figure 12:**
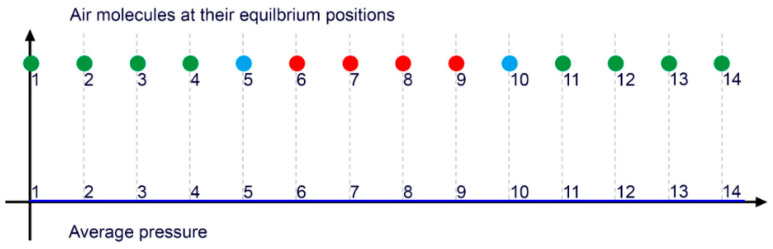
The particles of air are in equilibrium, evenly spaced in a random pattern (adapted from reference [26]).

**Analysis of IN VITRO Air Particles Movement**. At baseline, in a tube filled with air, the air particles remain undisturbed and occupy their equilibrium positions, distributed uniformly in a random configuration (Figure 12). When a pressure wave propagates, it adheres to fundamental acoustic principles. Rather than exerting force to drive or compress the entire column of particles, the wave only induces displacement of the particles from their equilibrium positions. This displacement occurs either axially, characterized by vertical oscillation at a fixed point, or longitudinally, involving oscillation along the length of the pipe [29].

**Figure 13:**
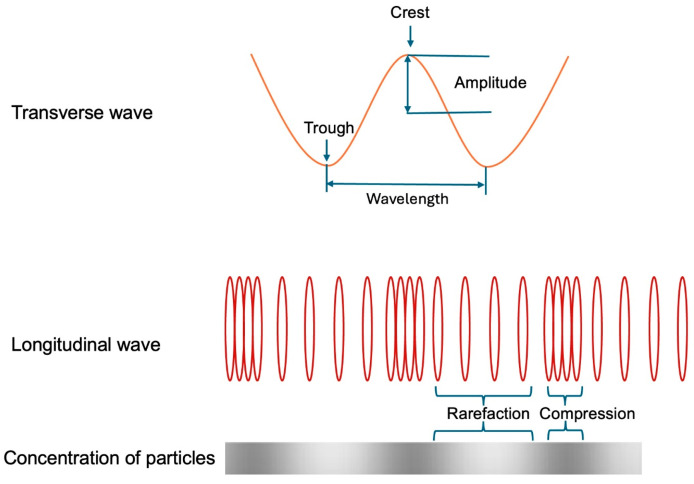
When a pressure wave passes by, the air particles are concentrated in zones of high density (compression), alternating with areas of moderate density (rarefaction). The zones of compression depict high pressure and turbulence. The zone of rarefaction has lower pressure fluctuation, and therefore, less turbulence.

**Mechanism of IN VITRO Particles Movement**. **Acoustic Perspective.** Once a pressure wave goes through, it generates alternating zones of particles dense concentration (compression) or moderate concentration (rarefaction). In the zones of compression, particles are driven inward from both sides toward a central point. Conversely, in the zone of rarefaction, particles are dispersed outward, moving away from the central point. In both cases, pressure fluctuates resulting in particles displacement either toward or away from a central point (Figure 13) [30].

**Figure 14:**
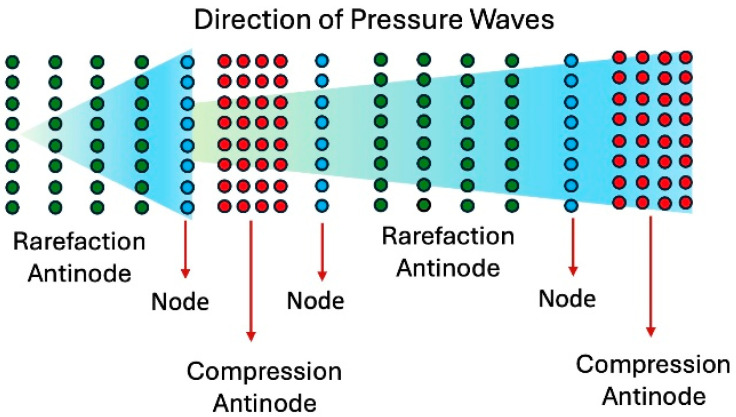
This is a conceptual schema of air particles when a pressure wave passes by, producing zones of high concentration of particles (compression) or moderate level of concentration (rarefaction). These zones of antinodes depict high pressure. The zone of nodes, which is located between the zone of compression and rarefaction, has no pressure fluctuation and a minimal concentration of particles [32,33,34].

In the context of movement dynamics, an antinode represents a point of maximal pressure variation (disorganized or turbulent), located at the peak of compression or rarefaction, where particle displacement is at its maximum. In contrast, a node represents a point of minimal pressure variation, situated at the junction between areas of compression and rarefaction. Here, the particle displacement is minimal, with little pressure turbulence (Figure 14) [31]. In this schematic image combining the movement of the particles based on their interaction with pressure, the main message is that the abrupt and chaotic change of pressure at the antinodes is the mechanism of damage in pipes and most likely in arteries.

**CRITICAL THINKING. Acoustic Effect on Air Particles in Tube and on Contrast Medium in Artery**. During a water hammer event, a pressure wave propagates retrogradely through the coronary artery, exhibiting characteristics analogous to a sound wave and traveling at velocities approaching sonic speeds. However, this phenomenon does not represent a true sound wave; instead, it is a pressure wave governed by acoustic principles.

As result, in the experiment of the air-filled tube, as the pressure wave passes through, even the particles move extensively, the high compressibility of air particles exhibits minimal internal pressure fluctuations. In contrast, in the artery with a liquid medium as blood, the pressure variations are significantly more pronounced, reflecting the incompressible nature of fluids.

**Figure 15:**
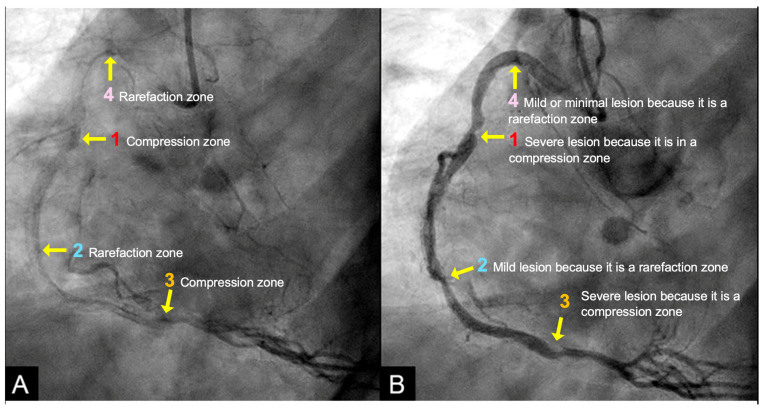
(**A**,**B**) This is the angiogram of the right coronary artery at the end of diastole with all the contrast almost flushed out. Pockets of high contrast concentration persist. These stagnation zones represent the high or moderate concentration of contrast particles and may correspond to zones of compression and rarefaction at the location of anti-nodes. The anti-node at **4** may define the distal end of the coronary artery as a tube based on the retrograde direction of the pressure wave. The anti-nodes at **1** and **3** may depict the zones of compression, while **2** and **4** may depict the zone of rarefaction. In between these antinodes, these coronary segments are the locations of nodes, which are clear of lesions because possibly there is no clash with the pressure wave.

**IN VIVO Coronary Flow Analysis. Angiographic Compression and Rarefaction Zones**. Building on an advanced understanding of pressure wave reflections in pipes from an acoustic perspective, this investigation applied acoustic principles to analyze flow dynamics in coronary arteries. The primary objective was to delineate and quantify regions within the coronary arteries characterized by pockets of high-contrast density (compression) and moderate-contrast density (rarefaction), identified as antinodes (Figure 15A,B). Conversely, areas with minimal contrast were designated as nodes. These classifications were finalized at the end of the second cardiac cycle when the contrast was almost all washed out, leaving pockets of contrast which most likely were zones of compression or rarefaction (labeled as antinodes). The needs for identifying the five zones of compression and rarefaction (antinodes) and the minimal or lesion-free segments (nodes) within a coronary artery are listed in Table 4.

**Figure 16:**
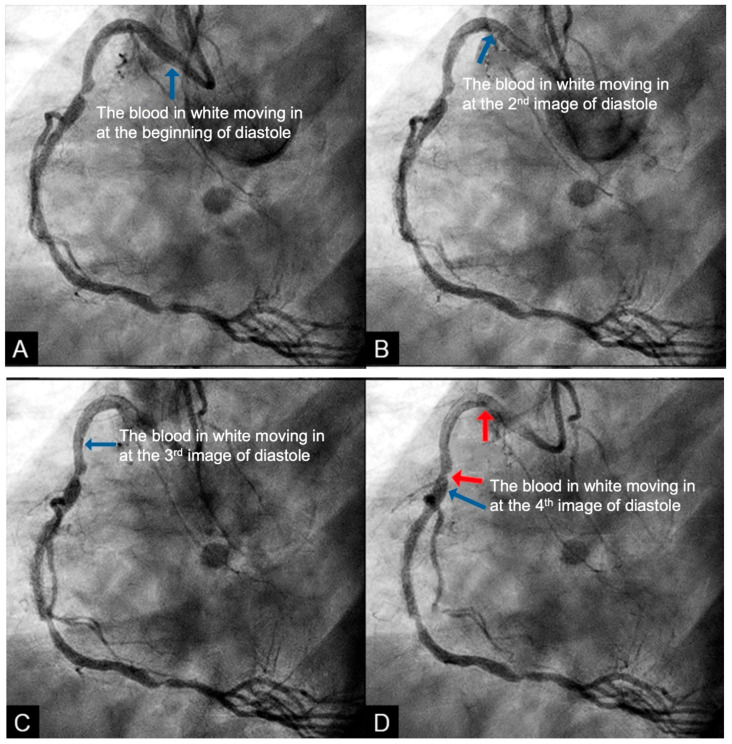

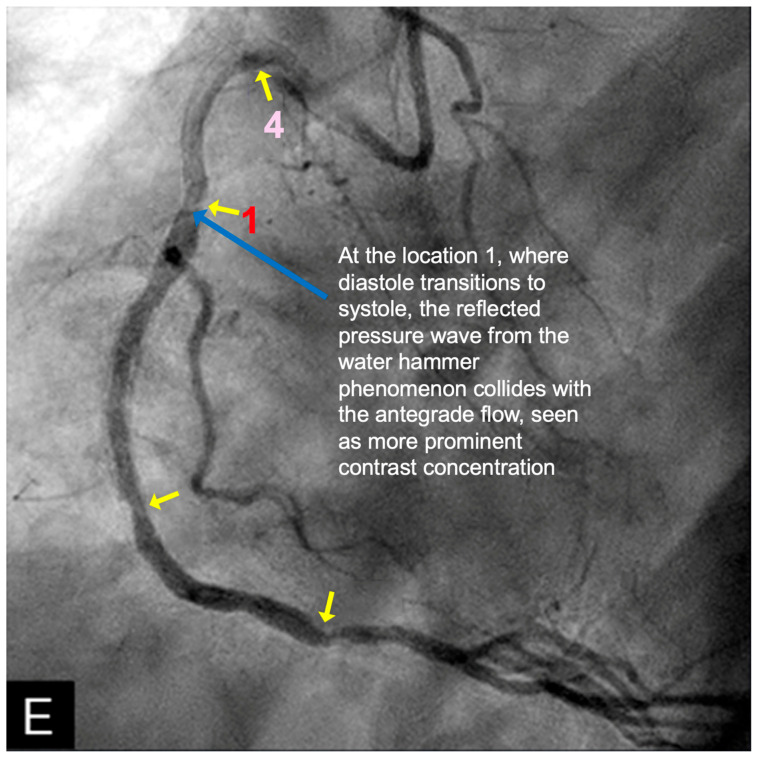
(**A**–**D**) These are four consecutive coronary images. (**A**) The blood in white begins to move in at the beginning of diastole (1D). (**B**) The blood in white is seen moving to the proximal segment of the right coronary artery (RCA) (2D). (**C**) The blood arrives at the center of the mid-segment of the RCA. (**D**) The blood in white is seen to advance a little more, reaching the lesion at the mid RCA. (**E**) This is the fifth image of the sequence from diastole to systole. From (**A**–**D**), the blood moves in rapidly in diastole. (**E**) The blood is stopped abruptly due to water hammer shock. The contrast concentration is more prominent at the location 1, where the retrograde pressure wave at the beginning of systole collides with the tip of the antegrade flow of diastole. The blue arrow shows a place where contrast is concentrated at the end of diastole. At the same time, the pressure wave reflects at the speed of sound, the anti-node 4 at the open end of the coronary artery could be seen early.

The secondary objective was to examine the relationship between antinodes and the presence of coronary stenoses, as well as between nodes and minimally diseased or lesion-free coronary segments. Ultimately, the investigation aimed to establish antinodes as potential markers for high-pressure surges linked to intimal damage. These locations of antinodes are used to construct a coronary acoustic activity (or action) map, enabling the identification of existing lesions, forecasting the progression of current lesions, and predicting the development of potential future lesions. Table 5 outlines the protocol on how to identify and label the five zones of compression and rarefaction (antinodes) and the minimal or lesion-free segments (nodes) within the coronary arteries. The protocol to identify the antinodes of compression and rarefaction and the nodes in between the antinodes uses the novel coronary dynamic angiographic technique. The protocol is listed in Table 5 and Figure 16A–E.

**FIRST Compression Zone with High Contrast Concentration. Angiographic Identification**. During diastole, coronary blood flow reaches a relatively high velocity. At the onset of systole, the contraction of the left ventricle abruptly interrupts this flow, triggering a water hammer effect. This results in the formation of a retrograde pressure wave, which collides with the antegrade flow at a critical timing when diastole transitions into systole. The initial reflection point, designated as location 1, is characterized by pockets of prolonged concentration of contrast agent, seen as a disorganized mixing of dark (contrast) and light (blood) materials (blue arrow in Figure 16E). These black-and-white pockets may signify turbulent flow, mirroring the surge of pressure in the local area (Figure 16A–D).

**Mechanism of Damage.** At location 1, damage mechanisms are likely driven by localized pressure spikes, stress concentrations, or repeated high-pressure cycles (vibrations), which induce deformation, cracking, or microtears in the intimal layer. These disruptions facilitate the migration of low-density lipoprotein (LDL) cholesterol into the subintimal space, initiating the atherosclerotic cascade (blue arrow in Figure 16E).

**Clinical Relevance**. The lesion at this location is very important because this is the most common lesion in the right coronary artery (RCA) and the most common location for ST elevation myocardial infarction in the RCA [35]. The reason is because this is the location where the pressure wave from water hammer hits first in its retrograde reflection, when diastole transitions to systole.

**CRITICAL THINKING. High Concentration of Contrast at location 1 as a Marker of Compression Activity**. During a typical cardiac cycle, antegrade blood flow accelerates during diastole and transitions rapidly into the systole, initiating a water hammer phenomenon that generates a retrograde pressure wave. This retrograde wave propagates at nearly the speed of sound and undergoes multiple reflections within one or two diastole–systole cycles along the length of a coronary artery. These reflections produce hundreds of retrograde pressure waves, which may synchronize with antegrade waves to form resonant patterns or, conversely, cancel out. The wave reflections occur at distinct locations: the diastole-to-systole junction (location 1), the coronary artery ostium (location 4), and the systole-to-diastole junction (location 2). The resulting wave dynamics display features of acoustic resonance, with regions of high contrast concentration corresponding to antinodes—pressure peaks associated with arterial damage or the progression of atherosclerosis (Figure 16E). High-contrast regions at locations 1 and 4, observed in coronary angiography, likely represent zones of compression and rarefaction, indicative of pressure surges that may compromise the intimal layer. The critical challenge lies in determining how these observations and hypotheses can be rigorously validated.

**Figure 17:**
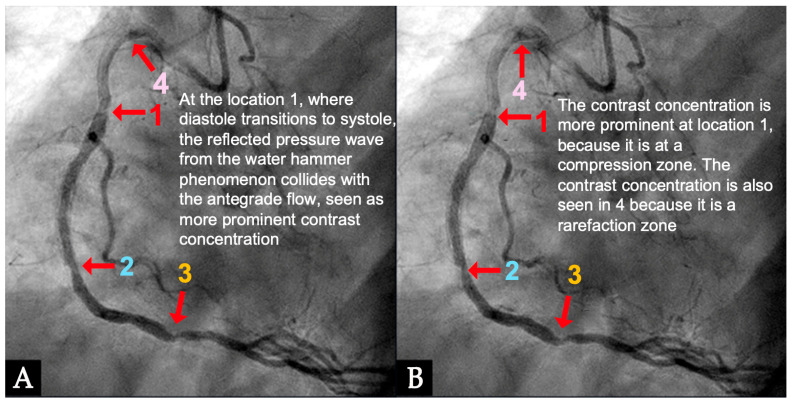

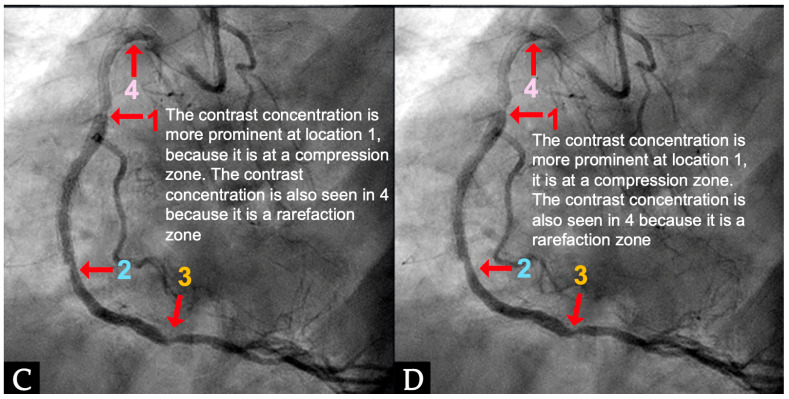
(**A**–**D**) This is the right coronary artery in mid-systole because the contrast in black is still seen at the distal segment. The concentration of dense contrast (antinode) persists at the mid segment (labeled as 1) and at the proximal segment (labeled as 4).

**SECOND Rarefaction Zone with Moderate Contrast Concentration Angiographic Identification.** Analogous to the retrograde propagation of a pressure wave caused by a water hammer toward the open end of a tube, when a high-density (compression) pressure wave reaches the ostial segment of a coronary artery—serving as an open interface—and contrast briefly displaces outward to equilibrate the pressure between the coronary artery and the aortic root. This displacement induces a localized pressure drop at the point of reflection. The resulting low-pressure zone, or rarefaction, propagates retrogradely through the coronary artery as a reflected rarefaction pulse (Figure 8 and Figure 17A–D). This phenomenon constitutes the second reflection point, labeled as 4 in Figure 16E, corresponding to the distal open end of the coronary artery, which is conceptualized as a tubular structure.

**Mechanism of Damage**. In the context of sound wave propagation, the antinode zone of rarefaction is characterized by the lowest pressure levels. Within this zone, the negative pressure during the rarefaction phase can lead to tissue stretching and deformation, potentially causing microtears or damage to the endothelial layer, which may initiate the atherosclerotic process [36]. Localized shear forces arising from rapid pressure fluctuations can further impair endothelial cell function, contributing to the development or progression of atherosclerotic plaques. The primary mechanisms of vascular injury in the rarefaction antinode zone include mechanical stress, vibration-induced tissue deformation, and shear forces, collectively fostering conditions conducive to atherosclerosis. Compared to the damage occurring at points of compression (from low to high pressure), the severity of injury in the rarefaction zone (from high to lower pressure) is notably lower [36].

**THIRD Rarefaction Zone with Moderate Contrast Concentration. Angiographic Identification.** At the onset of the cardiac cycle, antegrade flow is initiated during diastole. During systole, the contraction of the left ventricle generates a pressure wave, characteristic of the water hammer effect, which propagates retrogradely and impacts the leading edge of the antegrade flow, causing intimal injury at this location (labeled 1) where diastole transitions to systole. This pressure wave continues its retrograde propagation, ultimately reaching the ostium of the artery, identified as location 4. Simultaneously, coronary flow advances forward during systole, albeit at a reduced velocity. Upon the end of systole, diastole resumes. The transition point where coronary flow shifts from systole to diastole is identified as location 2 where the elevated systolic pressure gives way to the lower diastolic pressure.

**Mechanism of Damage.** At position 2, during the shift from systole to diastole, the interaction between the water hammer reflection wave and a region of reduced pressure induces a transient local pressure disturbance and generates a chaotic pressure gradient. This disruption accelerates particle motion within the flow, producing abrupt velocity fluctuations. These conditions foster turbulence, marked by irregular and chaotic particle trajectories, particularly when the interaction induces flow separation or intensifies shear stress along the vessel wall. The ensuing turbulence compromises the endothelial lining, serving as a catalyst for the initiation of the atherosclerotic cascade. However, because this is a transition from high pressure to lower pressure, the turbulence is not as powerful as in location 1, where the low pressure of diastole meets the high pressure of retrograde systole. As a result, the lesion at location 2 is not as severe as the one at location 1 (Figure 16E).

**CRITICAL THINKING. Advanced Acoustic Questions and Solutions.** In the search for the explanation of presence of coronary lesion by acoustics, the site of compression 1 experiences a notable pressure surge. However, how high is this pressure surge? Why does it take over 40 years of such pressure surges to induce intimal injury and develop a sizable atherosclerotic plaque? Furthermore, why are four antinodes and nodes observed in the right coronary artery, despite the wavelength of the acoustic pressure wave potentially exceeds 1000 meters (m) These questions are raised by clinician–scientists and cardiologists even though the hypothesis of acoustic interference influencing coronary flow appears highly plausible.

For the answer to the first question, at location 1, the antegrade diastolic flow is stopped abruptly by a pressure wave from a water hammer event triggered by the contraction of the left ventricle. The pressure surge is calculated based on the formula:

**ΔP_h_ = ρ ∗ c_s_ ∗ u**

where ΔP_h_ = increase of pressure due to water hammer (Pa); ρ = blood density (kg/m^3^) (=1.060 g/mL); c_s_ = velocity of sound in the fluid (m/s) = 1439 m/s for water (minor change for blood); u = coronary blood velocity (m/s) = 11–12 cm/s.

According to the formula, blood density and the velocity of sound in blood are constant and remain unaffected by interindividual variability. The primary variation lies in blood velocity between diastole and systole. Notably, the pressure surge is greater at the end of diastole at location 1 compared to the end of systole at location 2. This difference could provide insight into why the lesion at location 1 exhibits greater severity than the lesion at location 2 [37].

For the second question of the late effect of the pressure surge, the answer is that due to the short length of the coronary artery and its limited capacity for momentum transfer, pressure surges generate lower peak pressures compared to longer tubular structures [32]. Additionally, the cyclic contraction and relaxation of the left ventricle, occurring at a frequency of 60–80 beats per minute, create a continuous series of rapid incoming and reflected acoustic pressure waves within the coronary artery. Specifically, they manifest as high-frequency pressure oscillations due to rapid wave reflections over relatively short distances. These oscillations, characterized by low intensity and minimal pressure accumulation, contribute to the slow start of atherosclerotic plaques and their delayed development over several decades.

As the answer to the third question, given the short length of the coronary artery and the initiation and cessation of water hammer events with each left ventricular contraction, localized zones of compression and rarefaction are still observed at specific points where diastole transitions to systole and systole transitions to diastole. Despite the pressure wave generated by the water hammer effect having a wavelength exceeding 1000 m—over 100,000 times the length of the coronary artery—these zones persist due to dynamic interactions between coronary flow under the effect of the acoustic pressure wave.

**Figure 18:**
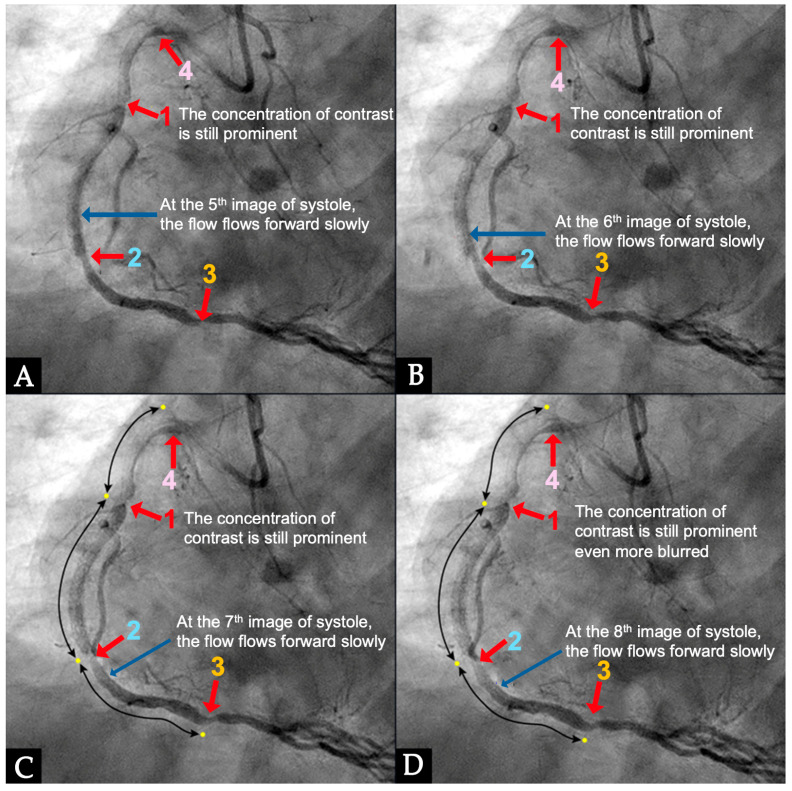
(**A**–**D**) This is the right coronary artery in mid-systole because the contrast in black is still seen at the distal segment. The concentration of dense contrast (antinode) persisted through segments. Between the locations of the antinodes (**1**, **2**, **3**, and **4**) the segments had no lesions or only minimal plaques. The reason is because at the locations of nodes, there was no high-pressure change, so no major damage of the intima was experienced.

**FOURTH No Lesion ZONE in the NODES.** In theory, the zones of nodes in a water hammer event experience minimal stress compared to the zones of antinodes, as nodes are characterized by stable pressure points with little fluctuation. These areas generally serve as neutral zones where the destructive effects of pressure surges are negligible. Since there is no significant pressure surge or high shear stress at the nodes, they are typically not sites of damage. Therefore, in the zones of these nodes, no stenosis or only minimal plaques or calcification with minimal lesion are identified (Figure 18C,D).

**CRITICAL THINKINGS. Pressure Surge and Mechanism of Damage.** When a pressure wave propagates through the blood of a coronary artery, it generates alternating regions of particle compression and rarefaction. At compression points (e.g., locations 1 and 3), there is a high concentration of contrast, whereas at rarefaction points (e.g., locations 2 and 4), the contrast concentration is less intense.

At location **1** with compression, corresponding to a pressure crest, the low-pressure antegrade flow during diastole is rapidly overtaken by a high-pressure surge during systole, producing a pronounced pressure peak. This abrupt surge induces disruption of the intima and increases the likelihood of severe damage leading to more severe lesions, plaque rupture, and the onset of acute coronary syndrome (ACS) (Figure 18A)

In contrast, at location **2** with rarefaction, representing a pressure trough, the transition de-escalating from high-pressure systolic flow to lower-pressure diastolic flow results in a pressure nadir. Unlike location **1**, the absence of substantial pressure surges at location **3** generates minimal turbulence, causing less intimal damage. As a result, lesions in these regions are less severe, and the risk of triggering ACS is comparatively lower. Furthermore, the pressure surge within the very short artery remains limited due to the short distance, preventing the accumulation of sufficient pressure momentum (Figure 18A,B).

At nodal points, where pressure fluctuations and particle motion are minimal, the lack of significant dynamic stress inhibits lesion formation and progression. Consequently, these nodal segments exhibit minimal or no lesions (Figure 18C,D).

**Figure 19:**
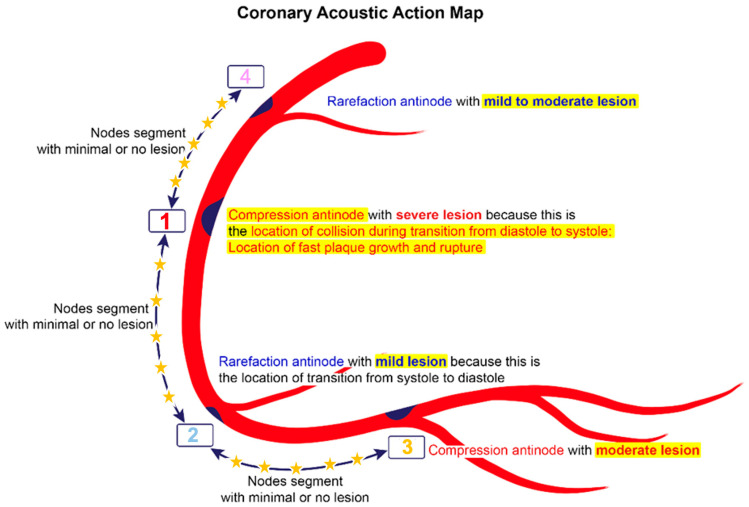
Coronary acoustic action map. In this right coronary artery (RCA), the antinodes with elevated turbulent pressure show strong correlation with lesion formation and progression. The most severe lesion happens at the compression antinodes (location 1 and 3), while less severe lesion happens at the rarefaction antinodes (2 and 4). Conversely, at the segments in between the antinodes (4-1, 1-2 and 2-3), regions with minimal pressure fluctuations show little to no lesion.

**Coronary Acoustic Action MAP.** The coronary flow investigations based on acoustics pinpointed pockets of high- or moderate-contrast concentrations, which might correspond to compression and rarefaction zones, respectively. Compression antinodes, linked to severe stenotic lesions, were believed to be due to rapid shifts from low-pressure diastolic flow to high-pressure systolic surges, causing turbulence and intimal disruption. Rarefaction antinodes, associated with milder lesions, were believed to be due to de-escalating transitions from high systolic pressure to lower diastolic pressure, resulting in less turbulence and milder injury. Nodes remained unaffected because there was no disorganized flow in its segments. An acoustic action map based on antinode and node locations could facilitate identifying current lesions and predicting future ones (Figure 19).

## 6. Clinical and Interventional Coronary Implications

**NEW Coronary Physiology and Pathophysiology Standards from Fluid Mechanics and Acoustics Perspective**. Under normal coronary dynamics, laminar flow constitutes the standard flow pattern, characterized by the smooth movement of the flow, without disturbances between the layers and absence of reversed flow or retrograde pressure waves associated with the water hammer phenomenon. This physiological state is typically observed in individuals with normal blood pressure and compliant arterial walls. Table 6 summarizes the clinical conditions arising from deviations in systolic and diastolic blood pressure, analyzed from a fluid mechanics and acoustics perspective.

**Figure 20:**
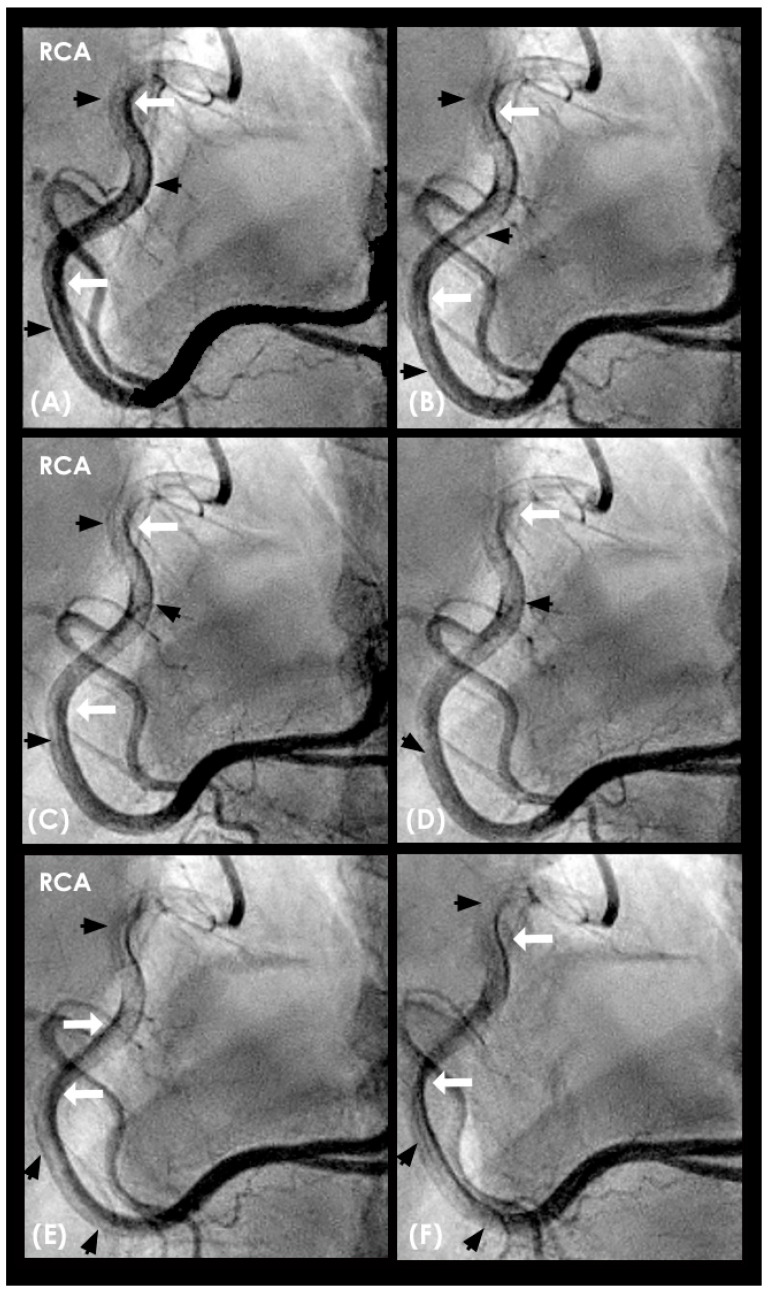
(**A**–**F**) **Laminar flow in right coronary angiogram**. (**A**–**D**) The blood (in white color) was observed moving along the apices of the curves (black arrowheads). The contrast (in black) with high viscosity occupied the inner curve (white arrows). (**E**,**F**) The blood in white is seen moving along the apices of the curves (three black arrows). The contrast in black with high viscosity occupies the inner curve. The contrast moves from one inner curve (first white arrow) to another inner curve (second white arrow). Laminar flow protects the intima from injury, so no lesion develops.

**CLINICAL APPLICATIONS Ideal Systolic and Diastolic Blood Pressure.** The first factor contributing to injury of the arterial intima is the nature of the blood flow. In young individuals with elastic coronary arterial walls and well-regulated blood pressure (systolic < 120 mmHg and diastolic < 80 mmHg), laminar flow progresses efficiently along the curvatures of the coronary arteries (Figure 20A–F). These conditions facilitate long-term cardiovascular stability and reduce the likelihood of development of coronary lesions [38]. This stability arises from the arterial wall’s capacity to absorb pressure surges, thereby attenuating pressure spikes that could otherwise damage the intima and initiate the atherosclerotic processes. Furthermore, maintaining low-density lipoprotein (LDL) cholesterol levels within recommended ranges is critical, as elevated total cholesterol—particularly in children with familial hypercholesterolemia—has been associated with early-onset coronary artery lesions [39].

**Figure 21:**
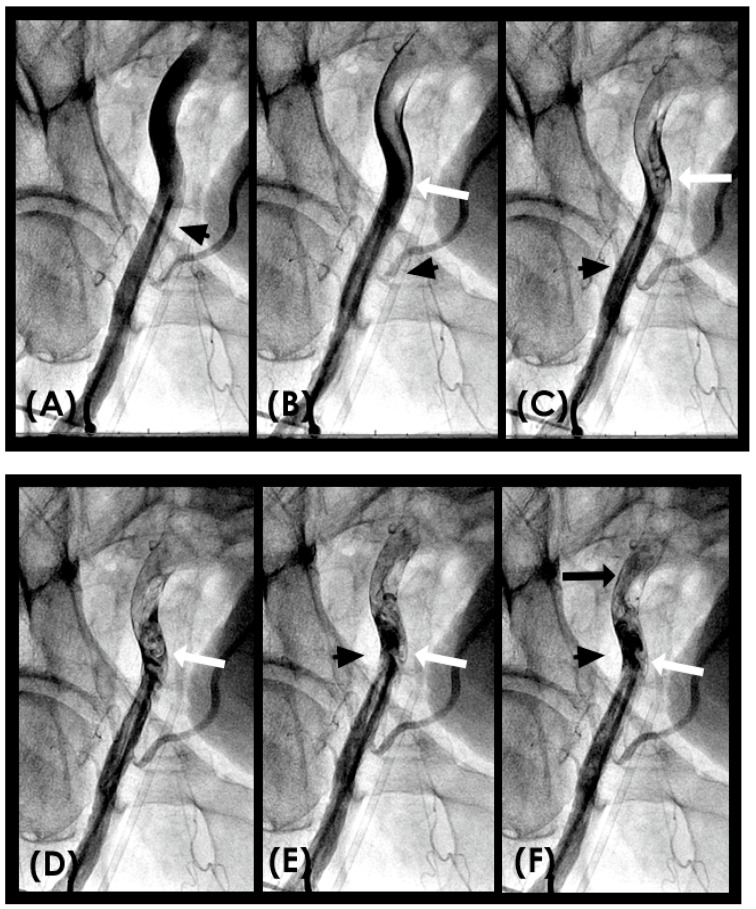
**Collision in the iliac artery.** This is a sequence of six consecutive angiographic images of the iliac artery. (**A**) The iliac artery is filled contrast in black. (**B**) Sixty-seven milliseconds later, the blood in homogenously white is seen moving down with a sharp tip of laminar flow (white arrow) (**C**) Subsequently, the pointed tip of the blood flow is halted abruptly, with all layers recoiling like a collapsing stack of dominoes (white arrow). (**D**) The tip of the flow then twists and turns on itself, resembling a vortex. (**E**,**F**) This turbulent vortical motion dissipates and is replaced by a mass of black contrast (arrowhead) moving in a retrograde direction along the inner curve (black arrow).

The second factor, from the perspective of fluid mechanics and acoustics, is blood pressure (BP). Blood pressure is regulated by two key components: systolic BP, determined by left ventricular contraction, and diastolic BP, influenced by peripheral vascular resistance. Elevated diastolic pressure in the peripheral arteries promotes retrograde flow in the iliac arteries, while high systolic pressure induces retrograde flow in the coronary arteries, leading to water hammer effects and subsequent retrograde pressure waves. Determining optimal systolic and diastolic BP thresholds to minimize retrograde flow and turbulence in the coronary and iliac arteries is critical. Based on the occurrence of retrograde flow, the ideal systolic BP is suggested to be <110 mmHg, while the optimal diastolic BP is <75 mmHg under conditions of minimal peripheral vascular resistance [40]. The reason is because retrograde flow in the iliac arteries is present and collides with the antegrade flow when diastolic BP is higher than 80 mmHg (Figure 21A–F)

**Figure 22:**
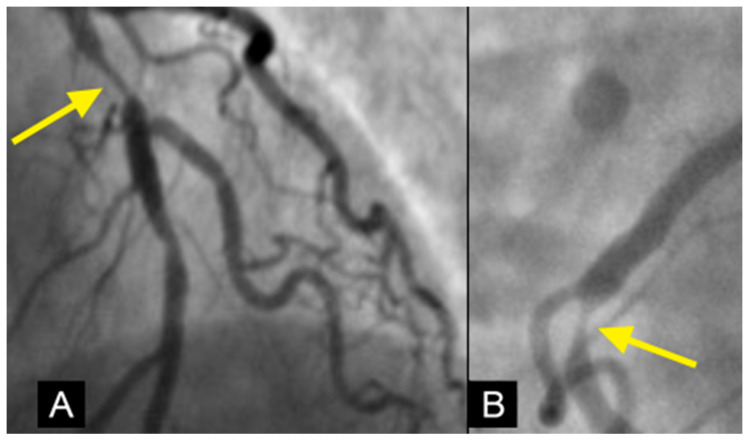
The left anterior descending (LAD) artery exhibits severe narrowing in its proximal segment (indicated by an arrow), while the right coronary artery (RCA) demonstrates a subtotal lesion in its mid-segment. In (**A**), the lesion in the proximal LAD appears to have resulted from damage inflicted by antegrade flow from uncontrolled diastolic hypertension, with the injury impacting the arterial wall and producing a characteristic “rat tail” appearance—featuring progressively severe narrowing in the distal direction. In (**B**), the mid-RCA lesion is attributed to damage from retrograde flow due to persistent uncontrolled systolic hypertension, which induced wall damage, leading to a “reversed rat tail” pattern, characterized by more severe narrowing proximally and a gradual reduction in severity distally.

**CLINICAL APPLICATIONS Differences in Management between Systolic versus Diastolic Hypertension.** In patients with uncontrolled high diastolic BP, the damage occurs in the antegrade direction due to rapid coronary antegrade flow during diastole. Under these conditions, the injury typically originates at the distal segment of the artery and gradually diminishes in severity toward the proximal segment, producing a lesion with a standard rat-tail configuration (Figure 22A). 

In patients with uncontrolled systolic blood pressure (BP), damage to the coronary artery arises from an abrupt water hammer effect, which generates a retrograde pressure wave. This retrograde wave leads to a collision with the antegrade flow at the transition from diastole to systole. This results in a lesion characterized by a reverse rat-tail configuration (Figure 22B). To provide personalized medicine, the patient of Figure 22A needs to control diastolic BP, while the patient in Figure 22B needs to have systolic BP under near perfect control [40].

**Figure 23:**
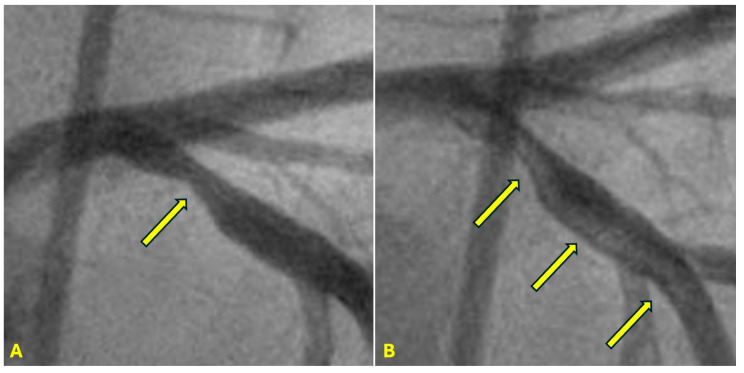
(A) The left anterior descending (LAD) artery exhibits mild to moderate narrowing in its proximal segment (indicated by an arrow). (**B**) This patient was interested in having optimal medical management with well-controlled blood pressure by betablockers and statins to decrease the low-density lipoprotein (LDL) cholesterol level < 75 mg%. There was laminar flow across the lesion (three yellow arrows in (**B**)). The patient has been stable for the last one year, without conversion to acute coronary syndrome.

**CLINICAL APPLICATIONS Progression or Regression of Intermediate Lesions.** Many patients with acute coronary syndrome (ACS) underwent coronary angiography, which revealed moderate lesions with haziness indicative of vulnerable plaques (Figure 23A,B). A critical question arises: should these patients undergo percutaneous coronary intervention (PCI), or should they receive optimal guideline-directed medical therapy (GDMT) instead? Furthermore, what diagnostic tools could effectively identify the risk factors that predispose plaques to vulnerability?

**Figure 24:**
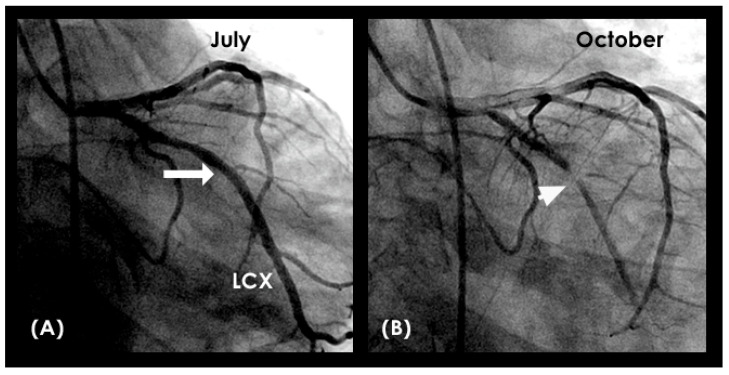

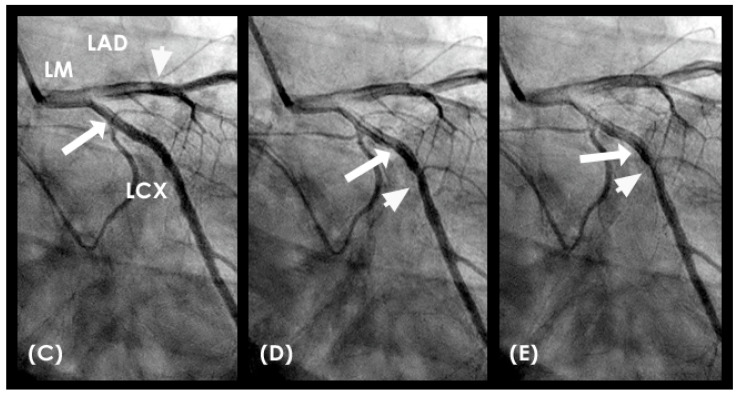
(**A**,**B**) **Left coronary artery angiogram**. (**A**) This is the left coronary angiogram with a patent left main (LM) and left circumflex (LCX) artery in a patient. (**B**) Three months later, a severe lesion in the mid-segment was observed (arrow). Could the physician have predicted the appearance of the severe lesion by reviewing the coronary flow of the July coronary angiogram? (**C**,**D**) The blood is seen moving in a proximal-to-distal segment at fast speed of diastole in laminar fashion with a thin boundary layer. (**E**) The blood flowed further downstream, but only at a minimal distance because this was the beginning of systole.

**Figure 25:**
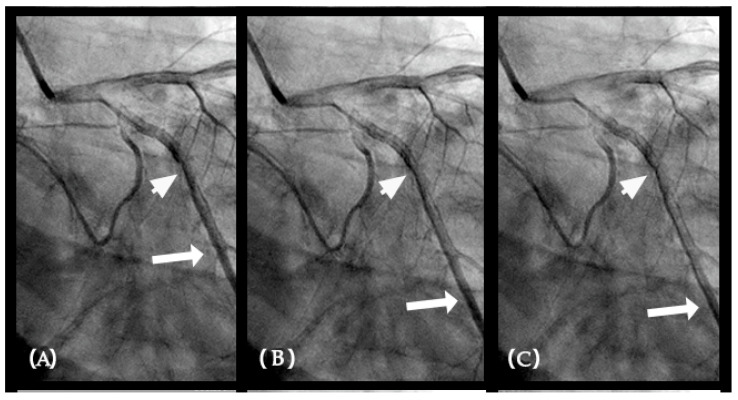
(**A**) The blood flowed further downstream (white arrow); however, there was a marked area with high concentration of contrast at the location transitioning from diastole to systole. This was the location of future lesion 3 months later (white arrowhead). This is the second image of the systole. (**B**) The high concentration of contrast (white arrowhead) continued at the location while the blood flow continued to flow forward distally (white arrow). This is the 3rd image of the systole. (**C**) The distal flow moved forward (black arrow, while there is some attenuation of the contrast (white arrow)). The flow looks more disorganized with a large boundary layer. This is the 4th image of the systole.

**Figure 26:**
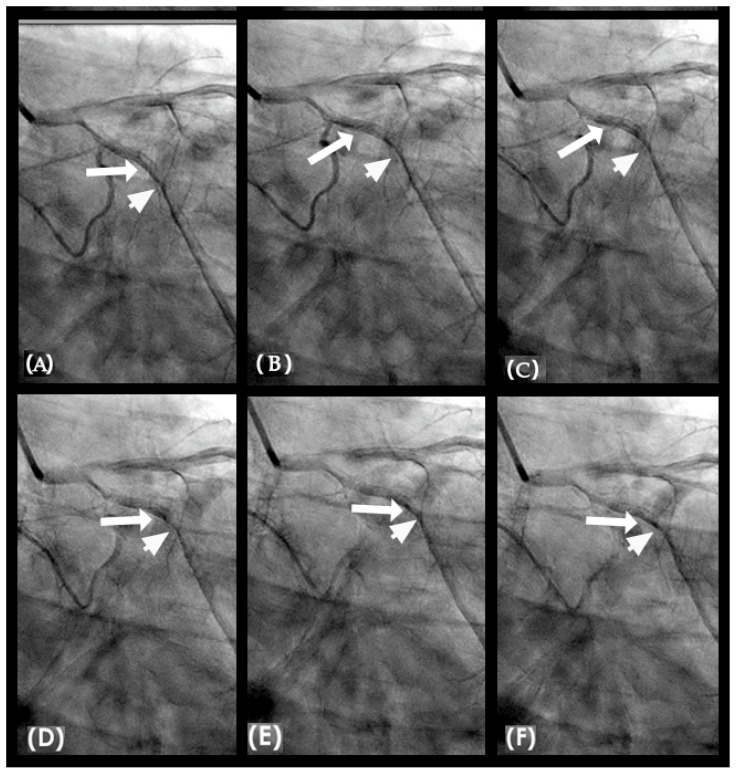
(**A**) The contrast seemed to fade away (white arrowhead) with a thin boundary layer (white arrow). This is the 5th image of the systole. (**B**) Black contrast is seen moving backward to the proximal left circumflex (white arrow). This was the angiographic evidence of reverse flow. (**C**) Black contrast is seen more prominently and moves backwards to the proximal left circumflex (white arrow). This is the angiographic evidence of reverse flow (white arrow). In the next 3 images (**D**–**F**), there is persistent stagnant contrast at the proximal segment of the LCX. This was the location of future lesion 3 months later (a little distal to the origin of the side-branch).

In dynamic coronary angiography, laminar flow is associated with the preservation of the structural integrity of pipe linings, pump components, and the intimal layers of coronary arteries. It plays a critical role in maintaining the stability of the cap cover of a plaque and the integrity of the endothelial cell layer. By inhibiting plaque progression, laminar flow contributes to the persistence of a stable angina (SA) condition, as demonstrated in the case above (Figure 23A,B). In contrast, turbulent flow damages the plaque, ruptures its cap, and precipitates ACS (Figure 24A–E, Figure 25A–C and Figure 26A–F) [41].

In patients with ACS, fractional flow reserve (FFR) may yield false-positive results due to flow obstruction caused by thrombi rather than atherosclerotic plaques and false-negative results because the plaque is vulnerable with an open crater, which can be covered by unstable layer of thrombi, which can be dislodged and trigger non-ST segment elevation myocardial infarction [41].

**CLINICAL APPLICATIONS Beneficial Effect of Stenting.** When a patient receives a stent, the technical goal is to deploy a stent with optimal expansion of the lumen and well apposition of the struts to the arterial wall. The fluid mechanics and acoustics goals are to develop a laminar antegrade flow during diastole, with no reversed flow, and with minimal boundary layers due to resistance from the metallic stent scaffold (Figure 11A–H). This restoration of laminar flow reduces the risk of both early thrombosis and late restenosis. The reason why there is no pressure wave reflection from a water hammer event is because the blood pressure is very well controlled during PCI, and the stented segment of the artery breaks the undulating movement of the arterial wall from pressure wave [42].

From a fluid mechanics perspective, the beneficial mechanism of stenting should not be merely described as opening a flow channel to the distal segment of the coronary artery. Instead, it is more accurately characterized as restoring laminar flow within the coronary artery and mitigating retrograde pressure waves associated with water hammer phenomena. This refined understanding extends the concept of beneficial mechanisms to include plain balloon angioplasty (POBA) and drug-coated balloon angioplasty (DCB) [43].

## 7. Conclusions

In the study of coronary artery disease (CAD), our new research direction conceptualizes the cardiovascular system as an integrated network of pumps and pipes, advocating for a paradigm shift from static imaging of coronary luminal stenosis to dynamic assessments of coronary flow. This analysis-combined-investigation review focused on the pressure wave reflections arising from the water hammer event and their detrimental effects on coronary flow dynamics.

The results showed that the flow investigations based on acoustics pinpointed pockets of high or moderate contrast concentrations, which most likely corresponded to compression and rarefaction zones, respectively. Compression antinodes were linked to severe stenotic lesions due to rapid shifts from low-pressure diastolic flow to high-pressure systolic surges, causing turbulence and intimal disruption. Rarefaction antinodes were associated with milder lesions due to de-escalating transitions from high systolic pressure to lower diastolic pressure, resulting in less turbulence and milder injury. Nodes remained void of lesions because of no pressure surge nor disorganized flow in its segments. Based on the locations of these antinodes and nodes, a coronary acoustic action map was constructed, enabling the identification of existing lesions, forecasting the progression of current lesions, and predicting the development of potential future lesions.

This review, integrating analysis and investigative findings, explores the potential link between water hammer-induced pressure dynamics and the initiation and progression of atherosclerotic lesions. By incorporating acoustic principles with advanced coronary flow imaging techniques to bedside management, a key preventive strategy for CAD involves sustaining laminar flow, which promotes long-term cardiovascular stability and reduces the risk of coronary lesion formation. This stability is achieved through the arterial wall’s ability to absorb pressure surges, thereby attenuating spikes that could otherwise compromise the intima and trigger atherosclerosis.

In patients with moderate lesions, laminar flow is also essential for maintaining the structural integrity of the fibrous cap, reducing the risk of plaque rupture. By suppressing plaque progression and rupture, laminar flow supports sustained coronary patency and lowers the likelihood of acute coronary syndrome. As a result, from a fluid mechanics perspective, the new change in strategy of medical treatment, balloon angioplasty, or stenting lies in reestablishing laminar flow within the coronary artery while mitigating retrograde pressure waves associated with the water hammer effect. If we achieve this goal, the eradication of CAD could be comprehensive and complete.

## 8. Limitations

The limitations of this study include its retrospective design, the use of a novel angiographic approach, and the presentation of only preliminary qualitative characteristics of coronary flow in a small patient cohort. Accordingly, the primary objective was to provide a scientific foundation for the potential occurrence of major fluid mechanics phenomena in coronary arteries, particularly the retrograde pressure wave associated with the water hammer effect, rather than to establish a definitive cause-and-effect relationship. The study’s methodological strength is inherently lower than that of large-population case–control prospective cohort studies or randomized clinical trials, where unmeasured confounding factors can be more effectively controlled.

## 9. Future Plan

This analysis-combined-investigation review serves only as the scientific foundation of the water hammer phenomenon in coronary arteries. In the future, there is a need to measure the pressure surge in compression and rarefaction antinodes, to prove the concentric movement of contrast at compression location and eccentric movement of contrast at rarefaction location, and to perform longitudinal study of lesions’ growth at the antinodes while sparing the segments of the nodes.

## 10. Perspective

**WHAT IS KNOWN?** Atherosclerosis is initiated by elevated wall shear stress in conjunction with hyperlipidemia, hypertension, and other contributing clinical risk factors.

**WHAT IS NEW?** The pressure surge caused by a retrograde pressure wave, as described in the water hammer phenomenon, results in localized injury to specific regions of the coronary artery, triggering the onset of the atherosclerotic cascade. Insights derived from fluid mechanics and acoustic principles suggest that effective management of atherosclerotic arterial disease should aim to reestablish standard laminar flow within the arteries and to abolish the pressure wave reflection of the water hammer phenomenon.

**WHAT IS NEXT?** These preliminary findings require validation through randomized clinical trials. The focus of the new hypothesis is to restore laminar flow with optimal medical management (well-controlled hypertension (systolic blood pressure < 120 mmHg and diastolic blood pressure < 80 mmHg and low-density lipoprotein (LDL) cholesterol < 75 mg%), by plain balloon angioplasty or drug-coated balloon (DCB) or stenting.

## Figures and Tables

**Table 1:** **Key dual fluid mechanics challenges in tubes and arteries.**

1. Identification of the damage and their mechanisms in pipes and arteries 2. Localization of lesion sites in pipes and arteries 3. Determination of boundary conditions in tubes and arteries 4. Analysis of the water hammer effect in short tubes and arteries 5. Comparison of the water hammer effect in flexible versus rigid tubes and arteries

**Table 2:** **Targets of the acoustic investigation of coronary flows.**

1. To identify presence or absence of **high contrast concentration** in the **main compression zone** (labeled as **1**) and correlation with presence or absence of lesion at **location 1** 2. To identify presence or absence of **moderate contrast concentration at the rarefaction zone** (labeled as **2**) and correlation with presence or absence of lesion at **location 2** 3. To identify presence or absence of **high contrast concentration** at the distal end of the coronary artery where the pressure wave is originated (labeled as **3**) and correlation with presence or absence of lesion at **location 3** 4. To identify presence or absence of **moderate contrast concentration (rarefaction zone)** at the proximal end of a coronary artery structured as a tube (labeled as **4**) and correlation with presence or absence of lesion at **location 4** 5. To identify presence or absence of the **NODES segment (bridging the compression and rarefaction zones)** and correlation with presence of only minimal or no lesion

**Table 3:** **Mapping location of current and future lesions.**

1. To identify the locations and parallels of air particle concentration in tube and contrast concentration in arteries 2. To identify the mechanisms and their similarity of air particles and contrast concentration in tube and arteries, respectively 3. To correlate pockets of contrast concentration in arteries to a location or a segment with or without lesions

**Table 4:** **Five coronary zones of high interest based on contrast concentration.**

1. **FIRST zone** of compression with high concentration of contrast, due to collision by the pressure wave against the tip of the antegrade flow (antinode labeled as 1). 2. **SECOND zone** of rarefaction with moderate concentration of contrast, due to the lower level of turbulence produced by the transition from high systolic pressure to diastolic pressure of a new cardiac cycle (antinode labeled as 2). 3. **THIRD Zone** of compression with high concentration of contrast, due to the collision of the pressure wave against the tip of the antegrade flow (antinode labeled as 3). 4. **FOURTH zone** of rarefaction with moderate concentration of contrast, marking the distal open end of a coronary artery structured as a tube (based on the retrograde direction of the pressure wave, antinode labeled as 4). 5. **FIFTH zone** of NODES which are positioned in the segments that bridge the boundaries of compression and rarefaction.

**Table 5:** **Protocol to identify the first antinode of compression.**

Locate the Main Compression Antinode (Labeled as 1) where the Diastole Transitions to Systole
1. First, the whole coronary artery is filled with contrast (Figure 16A). The recording is at 15 frames per second, or 0.067 milliseconds (msec) in between two consecutive images. 2. The tip of the antegrade blood flow (in white color) moves in. This is the first image of diastole (Figure 16B). 3. The blood moves in further. This is the 2nd image of the diastole (Figure 16C). 4. The blood moves further. This is the 3rd image of diastole (Figure 16D). 5. Here, at location **1**, the blood (in white) moves forward minimally because this is the end of diastole and beginning of systole. The blood (in white) stops abruptly due to a pressure wave from water hammer shock. The contrast concentration is more prominent at location **1,** where the retrograde pressure wave at the beginning of systole collides with the tip of the antegrade flow of diastole (blue arrow) (Figure 16E).

**Table 6:** **Management of coronary artery disease based on fluid mechanics and acoustic criteria.**

1. Definition of ideal systolic and diastolic pressure pre-empting any new formation or growth of coronary lesion 2. Different types of damage caused by uncontrolled systolic versus diastolic blood pressure 3. Treatment of hypertension: What is the best medication? Betablockers decrease the rate of rise, resulting in lower attenuation of the strength of water hammer 4. Effect of hypertension in the start of acute coronary syndrome (ACS): Laminar flow, no reverse flow (no water hammer), then no break-up of plaque and no progression to ACS 5. The beneficial effect of stenting is to restore the laminar flow and prevent retrograde pressure wave from the water hammer phenomenon

## Data Availability

The original contributions presented in the study are included in the article. Further inquiries can be directed to the corresponding author.
